# Brain DNA Methylation Atlas of *App^NL‐G‐F^* Alzheimer's Disease Model Mice Across Age and Region Reveals Choline‐Induced Resilience

**DOI:** 10.1111/acel.70241

**Published:** 2025-10-13

**Authors:** Andre Krunic, Thomas A. Bellio, Benjamin Z. Cohen, Adam Labadorf, Thor D. Stein, Honghuang Lin, Tiffany J. Mellott, Jan K. Blusztajn

**Affiliations:** ^1^ Department of Pathology and Laboratory Medicine Boston University Chobanian and Avedisian School of Medicine Boston Massachusetts USA; ^2^ Department of Pharmacology, Physiology, and Biophysics Boston University Chobanian and Avedisian School of Medicine Boston Massachusetts USA; ^3^ Department of Neurology Boston University Chobanian and Avedisian School of Medicine Boston Massachusetts USA; ^4^ Bioinformatics Program Boston University Boston Massachusetts USA; ^5^ VA Boston Healthcare System Boston Massachusetts USA; ^6^ VA Bedford Healthcare System Boston Massachusetts USA; ^7^ Boston University Alzheimer's Disease Research Center Boston University Chobanian and Avedisian School of Medicine Boston Massachusetts USA; ^8^ Department of Medicine University of Massachusetts Chan Medical School Worcester Massachusetts USA

**Keywords:** Alzheimer disease, choline, computational biology, DNA methylation, epigenomics, resilience

## Abstract

Alzheimer's disease (AD) is the most common type of dementia. Current treatments for AD are inadequate, and there is a need to design preventive strategies that would improve the resistance or resilience to AD pathology. Because aberrant brain DNA methylation (DNAm) is associated with hallmarks of AD, we tested the hypothesis that a nutritional approach using choline, an essential nutrient and methyl donor, would modulate DNAm to ameliorate AD pathologies. Previous studies showed that perinatal choline supplementation (PCS) reduced AD‐like neuropathology and inflammation while improving cognitive performance in AD mouse models. Here we investigated hippocampal and cerebral cortical DNAm patterns by reduced representation bisulfite sequencing from 3 to 12 months in wild‐type (WT) and *App* ^*NL‐G‐F*^ AD model mice fed a 1.1 g/kg control or 5.5 g/kg PCS diet from conception to weaning. *App* ^*NL‐G‐F*^ mice showed extensive CpG DNAm changes, which were associated with the age‐dependent progression of amyloidosis. PCS induced genotype‐specific DNAm patterns and reversed DNAm changes in multiple genes in *App* ^*NL‐G‐F*^ mice. By associating DNAm with matched transcriptomics, we found that DNAm in *App* ^*NL‐G‐F*^ mice correlated with the expression of microglial genes, while DNAm‐associated genes modulated by PCS were related to synaptic function. Moreover, we found that methylation levels of several CpGs were associated with levels of beta amyloidosis, relating epigenetic changes to neuropathology. Overall, our data suggest that DNAm in the brain serves as an epigenetic mechanism for abnormal gene expression in A*pp* ^*NL‐G‐F*^ mice and indicate that PCS may promote resilience to synaptic dysfunction through modulating DNAm.

## Graphical abstract

Brain DNA methylation drives abnormal gene expression in AppNL‐G‐F mice. Perinatal choline supplementation may promote resilience to synaptic dysfunction through modulation of DNA methylation.

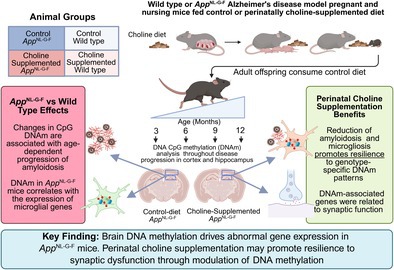

## 1 Introduction

Alzheimer's disease (AD) is a progressive age‐related neurodegenerative disease that affects over 50 million people worldwide and over 7 million in the US (*2025 Alzheimer's Disease Facts and Figures* 2025). The defining neuropathological features of AD are the accumulation of extracellular amyloid beta (Aβ) plaques and intraneuronal neurofibrillary tangles composed of hyperphosphorylated microtubule‐associated protein tau (Breijyeh and Karaman 2020). These pathological proteins stimulate inflammation through the engagement of microglia, astrocytes, and invading peripheral immune cells (Jorfi et al. 2023), leading to widespread synaptic loss, neurodegeneration, progressive cognitive impairment, and dementia. Current treatments for AD, including the recently introduced anti‐amyloid monoclonal antibodies, are inadequate (Belder et al. 2023) highlighting the need for the development of effective preventive measures for this illness.

Choline is an essential nutrient necessary for the proper functioning of all cells in the body. While a small amount of choline is generated endogenously, mostly by the liver, meeting the adequate intake (AI) recommendations requires obtaining it from the diet (Institute of Medicine (US) Standing Committee on the Scientific Evaluation of Dietary Reference Intakes and its Panel on Folate, Other B Vitamins, and Choline 1998; Zeisel and da Costa 2009). Choline is used for the synthesis of phosphatidylcholine (PC) and is required to synthesize acetylcholine (ACh), a neurotransmitter controlling muscle contraction, sympathetic nervous system activity, brain development, attention, learning, and memory. Following its enzymatic oxidation to betaine, choline can provide its methyl groups for the conversion of homocysteine to methionine and subsequently for the synthesis of S‐adenosylmethionine (AdoMet)—the source of methyl groups for most enzymatic methylation reactions (Blusztajn et al. 2017).

Previous studies have shown that PCS prevents age‐related memory decline in rodents, which spurred interest in its potential protective role in neurodegenerative diseases (Meck 2008). Indeed, PCS ameliorates many of the hallmarks of AD in animal models. In several mouse models of AD, PCS decreased amyloid burden by altering the processing of APP, reducing neuronal hyperexcitability, and attenuating NLRP3 inflammasome activation (Chartampila et al. 2023; Mellott et al. 2017; Wang et al. 2019). Recent evidence has shown that many of the benefits of PCS can be recapitulated through lifelong exposure (Velazquez et al. 2019). Conversely, lifelong choline deficiency increased soluble, fibrillar, and oligomeric Aβ42 levels in 3xTg mice while also exacerbating tau pathology (Dave et al. 2023). We previously found that in *App* ^*NL‐G‐F*^ AD model mice, PCS decreased brain amyloidosis and behavioral deficits compared to nonsupplemented counterparts (Bellio et al. 2024). In humans, low choline intake has been associated with increased dementia risk, whereas increased choline metabolism is associated with cognitive resilience in AD (Judd et al. 2023; Mathys et al. 2024; Yuan et al. 2022). While evidence for the benefits of PCS in AD pathology is clear, many of the molecular mechanisms underlying its effect are yet to be elucidated.

Due to the role of choline as a methyl donor via AdoMet, DNA methylation (DNAm) has emerged as a potential molecular mechanism by which PCS exerts its neuroprotective effects. DNAm is the most common epigenetic modification in mammals and predominantly occurs on the C5 position of cytosines of cytosine‐guanine dinucleotides (CpGs) (Moore et al. 2013). This process is catalyzed by DNA methyltransferase (DNMT) enzymes using AdoMet as a methyl donor (Moore et al. 2013). Proteins containing a methyl binding domain (MBD), such as methyl CpG binding protein 2 (MeCP2), can bind methylated CpGs in gene promoters to silence transcription; however, exceptions do exist (Moore et al. 2013; Wagner et al. 2014). In addition, DNAm may modulate the expression of distal genes, creating a system that establishes transcriptional networks (Aran et al. 2013; Cheishvili et al. 2017; Kennedy et al. 2018; Lancaster et al. 2022; Liu et al. 2023; van Eijk et al. 2012; Xu et al. 2019). Both PCS and maternal choline deficits have been shown to alter DNAm in offspring, suggesting the potential role of epigenetics in its protective functions (Davison et al. 2009; Korsmo et al. 2022; Kovacheva et al. 2007, 2009; Pauwels et al. 2017; Zeisel 2017). Numerous studies have implicated DNAm in the pathogenesis of AD (Nikolac Perkovic et al. 2021; Yokoyama et al. 2017). Global changes in 5‐mC levels have been reported in various brain regions in AD patients, with large‐scale hypomethylation observed in the hippocampus and vasculature (Chen et al. 2009; Chouliaras et al. 2013). Conversely, hypermethylation has been reported in the medial frontal and temporal gyri (Coppieters et al. 2014). Numerous genes are differentially methylated in the AD brain, such as *ANK1, BIN1, ABCA7*, and *HOXA*, as well as amyloidogenic pathway genes *APP and PSEN1* (De Jager et al. 2014; Fetahu et al. 2019; Gasparoni et al. 2018; Lunnon et al. 2014; Semick et al. 2019; Smith et al. 2018, 2019). Additionally, altered DNA methylation is associated with other pathological features of AD, including inflammation, gliosis, synaptic dysfunction, lipid metabolism, and tauopathy (Jeremic et al. 2023; Shireby et al. 2022; Weymouth et al. 2024).

In this study, we aimed to generate a comprehensive atlas of hippocampal and cerebral cortical DNA methylomes using reduced representation bisulfite sequencing (RRBS) at 3, 6, 9, and 12 months of age in WT and AD model *App* ^*NL‐G‐F*^ mice following a control or PCS diet. The *App* ^*NL‐G‐F*^ mice exhibit progressive Aβ42 plaque accumulation as early as 2 months, with subsequent neuroinflammation and deficits in synaptic function (Latif‐Hernandez et al. 2020; Nilsson et al. 2014; Saito et al. 2014). We identified thousands of genotype‐ and diet‐associated DNA methylation differences in the brain and integrated RRBS data with bulk RNA‐seq results from our companion study (Bellio et al. 2025) to identify expression‐associated CpGs that were changed by genotype or diet. In addition, we integrated DNAm with amyloid plaque staining and found an association between amyloidosis and epigenetic changes. PCS prevented multiple brain methylomic and transcriptomic abnormalities in *App* ^*NL‐G‐F*^ mice.

## 2 Results

### 2.1 *App* ^*NL*^ ^*‐G‐F*^ Mice Show Altered DNA Methylation in Association With Age, Sex, and Brain Region Compared to WT

**FIGURE 1:**
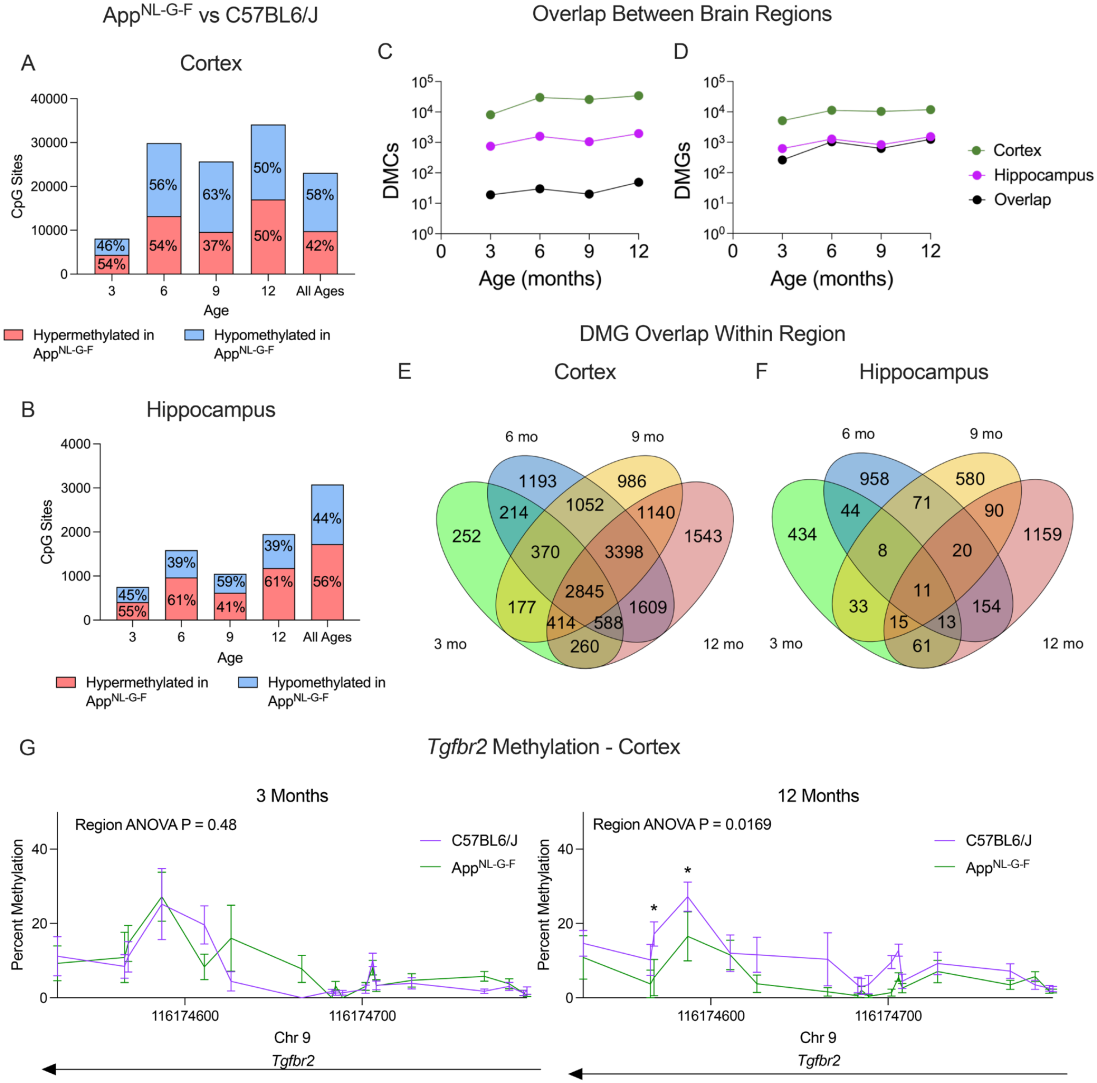
*App* ^*NL‐G‐F*^ mice show age and brain region‐dependent DNA methylation changes compared to wild‐type. Reduced representation bisulfite sequencing was performed on the hippocampus and cortex of male and female *App* ^*NL‐G‐F*^ or C57BL/6J mice at 3, 6, 9, and 12 months reared on a control diet (*n* = 3 per age, genotype, and sex, *n* = 48 total). CpGs with < 0.05 q‐value were considered differentially methylated. Distribution of differentially hyper‐ and hypomethylated cytosines (DMCs) at each age in (A) cortex and (B) hippocampus, controlling for sex and cell type proportion. (C) Overlap of DMCs in the cortex and hippocampus over time. (D) Overlap of differentially methylated genes (DMGs) in the cortex and hippocampus over time. Overlap of DMCs in (E) cortex and (F) hippocampus based on age. (G) Comparison of methylation between *App* ^*NL‐G‐F*^ or C57BL/6J in the promoter of *Tgfbr2* at 3 and 12 months. Data shown as mean ± SEM. *FDR < 0.05.

DNA methylation patterns differed in *App* ^*NL‐G‐F*^ mouse cortex and hippocampus as compared to the WT mice at all ages across the genome, controlling for age, sex, and cell type proportion (Figures 1A,B and S1). Cell proportion was determined via DecompPipeline, where we were able to identify relative cell type proportions that change in *App* ^*NL‐G‐F*^ and WT mice, including neurons and glia (Figure S2). Overall, the genome of the *App* ^*NL‐G‐F*^ mice tended to be hypomethylated in the cortex and hypermethylated in the hippocampus as compared to WT (Figure 1A,B, *n* = 6, FDR < 0.05). However, in the cortex of the *App* ^*NL‐G‐F*^ mice, there was a high density of hypomethylated CpGs on the X chromosome as compared to the WT mice (Figure S1). This was not observed in the hippocampus. There was limited overlap in genotype‐related differentially methylated CpG sites (DMCs) between the cortex and hippocampus across all ages (Figure 1C). This result was not due to insufficient CpG coverage, as most sites were covered in both regions (Figure S3). However, when mapping each CpG to its nearest gene, we observed that a high proportion of differentially methylated genes (DMGs) in the hippocampus were also differentially methylated in the cortex (3mo = 42%, 6mo = 81%, 9mo = 75%, 12mo = 82%, Figure 1D). This indicates that the methylation of similar genes is affected by the *App* ^*NL‐G‐F*^ genotype, but that these genes harbor somewhat different methylation patterns in each brain region, suggesting region‐specific gene regulation.

The number of DMGs in the cortex at 3 months of age was 5120, and higher at older ages (11,269, 10,382, and 11,799 at 6, 9, and 12 months, respectively, Figures 1E and S4). However, over 50% (2845) of the DMGs at 3 months remained differentially methylated at all ages, indicating that DNAm abnormality in *App* ^*NL‐G‐F*^ mice emerges early and is maintained at the gene level as the neuropathology progresses, while additional age‐related abnormalities continue to accumulate. This relationship was not observed in the hippocampus, which had many fewer DMGs as compared to the cortex, and over 70% of those were unique at each age (Figure 1F). Microglial receptor *Tgfbr2* is an example of one such gene in the cortex that over time becomes hypomethylated in *App* ^*NL‐G‐F*^ mice, in line with the progression of microgliosis (Figure 1G).

As DNAm is a key component in genetic imprinting, we investigated the presence of DMCs within imprinted genes (Tucci et al. 2019) and found multiple imprinted genes that were differentially methylated in *App* ^*NL‐G‐F*^ mice compared to WT (Figure S5A,B). Moreover, several imprinted genes in the cortex were also differentially expressed, including TAM receptor *Axl* and *Igf2r*, a process known to be regulated by DNAm (Figure S5).

We further compared the cortical methylome of *App* ^*NL‐G‐F*^ mice to previously published human methylation data (Piras et al. 2023; Semick et al. 2019; Shireby et al. 2022; Smith et al. 2021; Wang et al. 2023; Zhang et al. 2020). We found close to 44% of differentially methylated genes in human AD were shared in our model in the cortex and hippocampus (Supporting Information File S1), suggesting the *App* ^*NL‐G‐F*^ model can accurately recapitulate many epigenetic changes seen in human disease.

Given that AD neuropathology is sexually dimorphic (Ferretti et al. 2018), we stratified the subjects by sex and examined DMCs in *App* ^*NL‐G‐F*^mice as compared to WT mice. The autosomes of male and female *App* ^*NL‐G‐F*^ mice were characterized by hypermethylated DNA as compared to WT mice, with males showing a greater number of DMCs at later ages in both brain regions examined (Figure S6). Because the process of X chromosome inactivation in females includes high levels of DNA methylation of the inactive chromosome, the overall average DNA methylation of the X chromosome was indeed higher in females as compared to males. In contrast to the autosomes, the X chromosome tended to be hypomethylated in the cortex of *App* ^*NL‐G‐F*^ mice as compared to WT mice. This was particularly apparent in 3‐month‐old males and 9‐month‐old females (Figure S7A,B,E,F). In the hippocampus, there were fewer DNA methylation differences between the two sexes (Figure S7C,D).

### 2.2 Perinatally Choline‐Supplemented *App* ^*NL*^ ^*‐G‐F*^ Mice Show Altered DNA Methylation Compared to *App* ^*NL*^ ^*‐G‐F*^ Mice Fed a Control Diet

**FIGURE 2:**
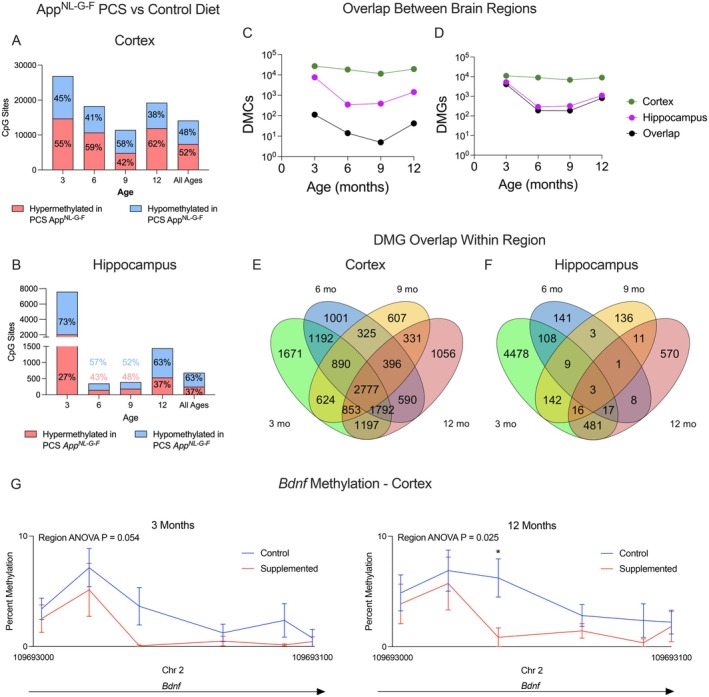
*App* ^*NL‐G‐F*^ mice fed a perinatal choline‐supplemented diet show age and brain region‐dependent DNA methylation changes compared to the control diet. Reduced representation bisulfite sequencing was performed on the hippocampus and cortex of male and female *App* ^*NL‐G‐F*^ mice reared on either a perinatal choline‐supplemented (PCS) or control diet at 3, 6, 9, and 12 months (*n* = 3 per age, diet, and sex, *n* = 48 total). Brain regions were taken from the same animal and were treated as within‐subjects. CpGs with < 0.05 q‐value were considered differentially methylated. Distribution of differentially hyper‐ and hypomethylated cytosines (DMCs) at each age in (A) cortex and (B) hippocampus, controlling for sex and cell type proportion. (C) Overlap of DMCs between *App* ^*NL‐G‐F*^ mice fed a PCS and control diet. (D) Overlap of differentially methylated genes (DMGs) between *App* ^*NL‐G‐F*^ mice fed a PCS and control diet. Overlap of DMCs in (E) cortex and (F) hippocampus based on age. (G) Comparison of methylation between the PCS and control diet *App* ^*NL‐G‐F*^ mice in *Bdnf* at 3 and 12 months. Data shown as mean ± SEM. *FDR < 0.05.

We characterized the effect of a PCS diet on the methylomes of *App* ^*NL‐G‐F*^ and WT mice. PCS *App* ^*NL‐G‐F*^ mice had altered global DNA methylation patterns in both the hippocampus and cortex compared to genotype‐matched control diet mice, with the greatest number of DMCs found at 3 months in both regions (Figures 2A,B and S8, *n* = 6, FDR < 0.05). PCS WT mice showed global methylation changes, and a high degree of gene‐level methylation overlap with supplemented *App* ^*NL‐G‐F*^ when compared to their respective genotypes fed a control diet in the cortex, but not the hippocampus (Figure S9). As compared to the *App* ^*NL‐G‐F*^ mice fed the control diet, *App* ^*NL‐G‐F*^ mice fed a PCS diet had multiple DNA methylation changes across all time points in the cortex, trending towards overall hypermethylation (Figure 2A). However, in the hippocampus, *App* ^*NL‐G‐F*^ mice fed a PCS diet had a large degree of hypomethylation at 3 months that dropped greatly by 6 and 9 months (Figure 2B). There was limited overlap in DMCs due to the PCS diet in *App* ^*NL‐G‐F*^ mice between the cortex and hippocampus across all ages (Figure 2C). The lack of concordance between brain regions was not due to insufficient overlap in coverage between the two brain regions (Figure S10). However, the majority of DMGs in the hippocampus were also differentially methylated in the cortex, suggesting the same genes are differentially methylated at different CpGs (3mo = 78%, 6mo = 66%, 9mo = 58%, 12mo = 72%, Figure 2D). Up to 25% of cortical DMGs at 3 months remained differentially methylated until 12 months (Figure 2E), with many time‐specific DMGs. The hippocampus showed little overlap in DMGs over time (Figure 2F). This lack of overlap was not due to disparities in coverage (Figure S11). One notable region that was differentially methylated in the cortex is a promoter region of the key neurotrophic factor *Bdnf*, which is consistently hypomethylated in PCS *App* ^*NL‐G‐F*^ mice relative to the control diet (Figure 2G).

We further investigated imprinted genes whose methylation in *App* ^*NL‐G‐F*^ mice was altered by perinatal diet (Figure S12). Interestingly, PCS caused alterations in imprinted genes in the cortex at all ages, while in the hippocampus, most imprinted genes were changed only at 3 months. Additionally, several imprinted genes also showed altered gene expression by PCS, including *Magi2* in the cortex and *Cd81* in the hippocampus (Figure S12).

### 2.3 Perinatal Choline Supplementation Can Reverse Genotype‐Induced DNA Methylation Changes in *App* ^*NL*^ ^*‐G‐F*^ Mice

Given that PCS can alter DNAm patterns in *App* ^*NL‐G‐F*^ mice, we compared DMCs that were changed by both genotype and choline supplementation. When controlling for age, we identified DMCs in the cortex and hippocampus (Figure S13A,B) that were altered by both *App* ^*NL‐G‐F*^ genotype and PCS. PCS reversed genotype‐dependent methylation changes of these CpGs in both cortex and hippocampus (Supporting Information File S2, Figure S13C,D). We further identified genes that were differentially methylated in both treatment groups and saw a notable overlap. Interestingly, in the sites and genes that were shared between groups, PCS consistently reversed the effect of *App* ^*NL‐G‐F*^ genotype (Figure S13E–H). As an example from the cortex, methylation of chr8:46540075, located near *Acsl1*, is decreased in *App* ^*NL‐G‐F*^ mice at 9 months and restored to WT levels by PCS (Figure S13I). *Acsl1*‐positive microglia have been previously linked to lipid droplet accumulation in AD (Haney et al. 2024). In the hippocampus, the methylation of chr10:94052312, located within *Fgd6*—a gene whose polymorphism is associated with healthspan and longevity in humans—shows sustained hypomethylation in *App* ^*NL‐G‐F*^ mice compared to WT that was reversed by choline supplementation (Figure S13J) (Timmers et al. 2020).

### 2.4 DNA Methylation Changes in *App* ^*NL*^ ^*‐G‐F*^ Mice Are Associated With Altered Gene Expression

**FIGURE 3:**
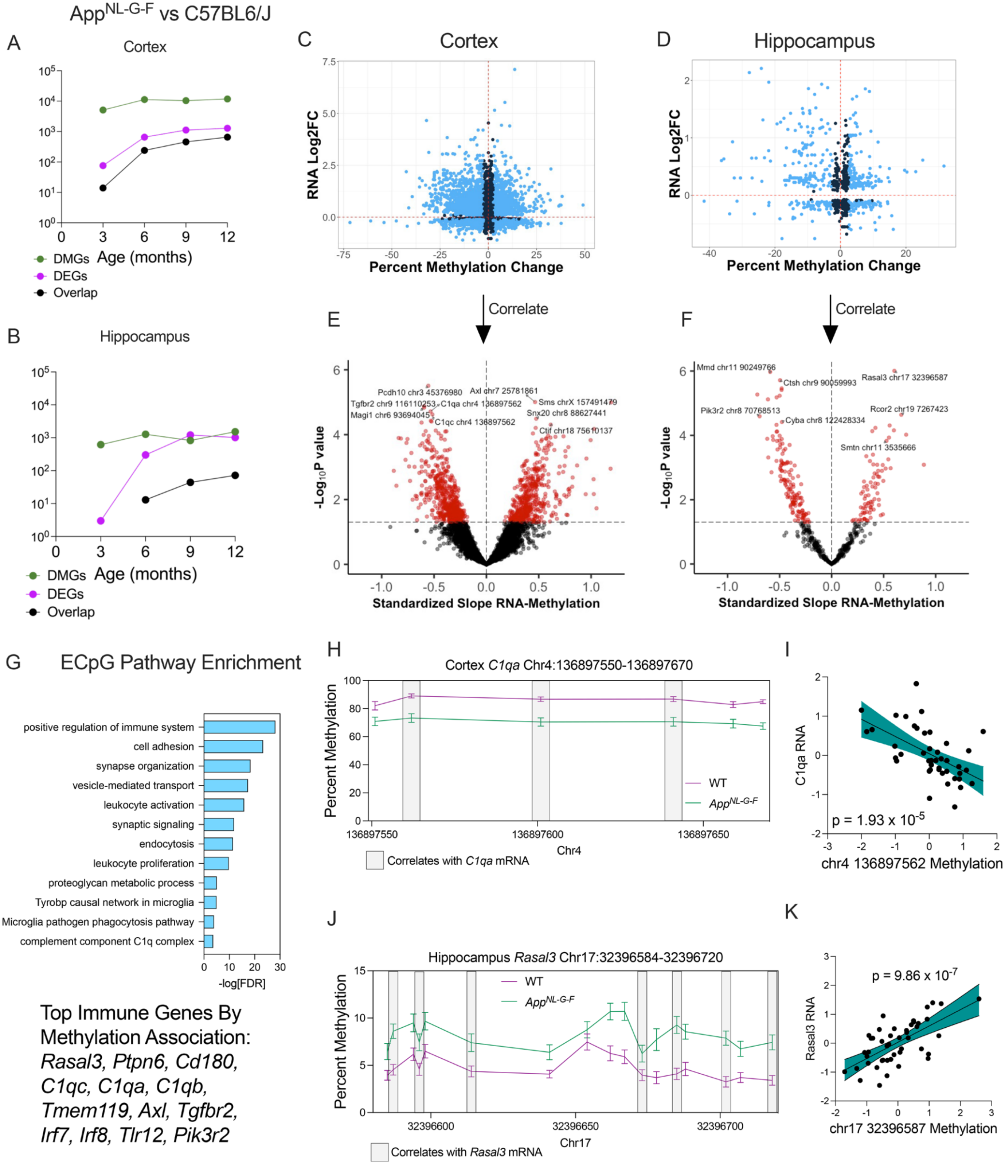
DNA methylation changes in *App* ^*NL‐G‐F*^ mice are associated with local changes in transcription. DMCs previously identified were mapped to their nearest gene and associated with transcription from matched bulk RNA‐seq data. DEGs from RNA‐seq were defined as FDR < 0.05. DEGs and DMGs controlling for sex and cell composition (DMGs only) between *App* ^*NL‐G‐F*^ and WT mice fed a control diet were compared in (A) cortex and (B) hippocampus to identify differentially methylated and expressed genes. (C) DEG–DMC pairs in the cortex show a negative correlation between methylation and gene expression (r = −0.08, *p* < 2.2 × 10^−16^). (D) DEG‐DMC pairs in the hippocampus show a negative correlation between methylation and gene expression (r = −0.21, *p* = 7.0 × 10^−10^). DEG–DMC pairs with above a 2.5% change in methylation and 0.1 log2 fold change are highlighted for downstream analysis. Linear regression between RNA counts and methylation, controlling for sex, age, and cell composition was performed in (E) cortex and (F) hippocampus on pooled DEG–DMC pairs from each age group. (G) Pathway enrichment of methylation‐associated genes differentially expressed in *App* ^*NL‐G‐F*^ mice across both brain regions (Top immune‐system‐associated genes are listed). (H) Example of a DMR changed in the cortex within the *C1qa* gene. (I) Added variable plot of Z‐scaled values of chr4:136897562 methylation and *C1qa* expression (*n* = 43, β_methylation_ = −0.53, *p* = 1.93 × 10^−5^). (J) Example of a DMR changed in the hippocampus within the *Rasal3* gene. (K) Added variable plot of Z‐scaled values of chr17:32396587 methylation and *Rasal3* expression (*n* = 46, β_methylation_ = 0.60, *p* = 9.86 × 10^−7^).

To determine the potential functional roles of DNAm, we integrated our RRBS data with bulk RNA sequencing results from the same tissue in our complementary study (Bellio et al. 2025). To identify expression‐linked CpGs (ECpGs), we assessed the overlap between differentially expressed genes (DEGs) and the nearest DMCs. In the cortex of *App* ^*NL‐G‐F*^ mice versus WT mice fed a control diet, we identified numerous DEGs, and their number increased with mouse age in the cortex (Figure 3A) and hippocampus (Figure 3B). Interestingly, we found methylation changes in *App* ^*NL‐G‐F*^ cortex and hippocampus at 3 months, which predate notable transcriptomic changes (Bellio et al. 2025). Mapping individual DEG‐DMC pairs confirmed a trend toward increased methylation in downregulated genes (Figure 3C,D). Blue points represent DEG–DMC pairs that show both at least a 0.1 log_2_ fold change in expression and 2.5% change in methylation between *App* ^*NL‐G‐F*^ and WT animals, which were further investigated in downstream analysis.

CpG‐Gene pairs from each time point were then pooled and tested for association by linear regression between percent methylation and RNA expression. We identified 1032 CpG‐mRNA pairs (Figure 3E) in the cortex and 217 CpG‐mRNA pairs in the hippocampus (Figure 3F) that were significantly correlated (Supporting Information File S3). The expression of 40 genes was associated with CpG methylation in both brain regions. These include key microglial genes such as *C1q, Sall1, Irf8*, and *Csf2ra*. Over‐representation analysis of methylation‐associated genes across both regions revealed an enrichment of pathways implicated in AD pathological processes, including microglial activation and synaptic function (Figure 3G).

In both brain regions, ECpGs tended to cluster as DMRs. One example in the cortex was *C1qa*, a component of the complement pathway that has been associated with Aβ plaque phagocytosis (Webster et al. 2000) and has been designated as a plaque‐induced gene (PIG) (W.‐T. Chen et al. 2020). Part of the *C1qa* gene contains a DMR that is hypomethylated in *App* ^*NL‐G‐F*^ mice (Figure 3H). However, while multiple sites are differentially methylated, only some CpGs are associated with gene expression, such as chr4:136897562 (*n* = 43, Standardized β_methylation_ = −0.53, *p* = 1.93 × 10^−5^, Figure 3I). In the hippocampus, there was a 136 bp DMR located within the *Rasal3* gene that was hypermethylated in *App* ^*NL‐G‐F*^ mice (Figure 3J). This DMR is located within the gene body and has a positive correlation with expression, as shown by the example chr17:32396587 (*n* = 46, Standardized β_methylation_ = 0.60, *p* = 9.86 × 10^−7^, Figure 3K). *Rasal3* is involved in inflammation in the periphery and its expression is upregulated in *App* ^*NL‐G‐F*^ mice, but its role in the brain is currently unknown (S. Saito et al. 2021).

### 2.5 Methylation Changes in *App* ^*NL*^ ^*‐G‐F*^ Mice Fed a Perinatal Choline Supplemented Diet Are Associated With Changes in Gene Expression

**FIGURE 4:**
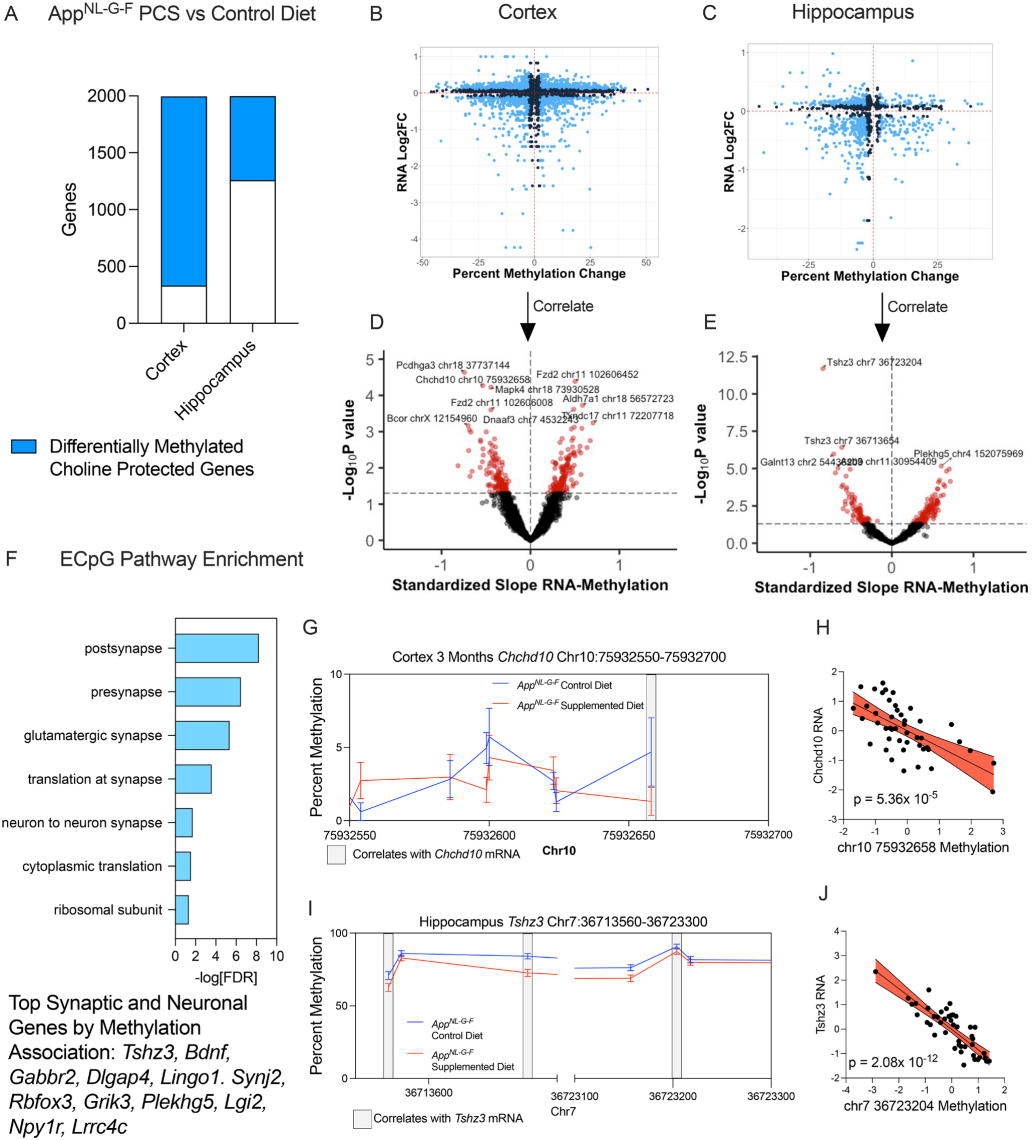
DNA methylation changes in choline‐supplemented *App* ^*NL‐G‐F*^ mice are associated with local changes in transcription. DMCs previously identified were mapped to their nearest gene and associated with transcription from matched bulk RNA‐seq data. Choline‐protected genes were defined as genes differentially expressed between *App* ^*NL‐G‐F*^ and WT mice fed a control diet, but not *App* ^*NL‐G‐F*^ and WT mice fed a PCS diet. Choline‐protected genes and DMGs in *App* ^*NL‐G‐F*^ mice fed a choline‐supplemented or control diet were compared in (A) cortex and hippocampus to identify differentially methylated and expressed genes. DEG–DMC pairs in the (B) cortex and (C) hippocampus plotted by percent methylation change and RNA fold change. Linear regression correlation coefficients between RNA counts and methylation, controlling for sex, age, and cell composition in (D) cortex and (E) hippocampus DEG‐DMC pairs. (F) Pathway enrichment of methylation‐associated genes differentially expressed in *App* ^*NL‐G‐F*^ PCS mice across both brain regions (Top synaptic and neuronal genes are listed). (G) Example of a DMC changed in the cortex within the *Chchd10* gene at 3 months (*n* = 6). (H) Added variable plot of Z‐scaled values of chr10:75932658 methylation and *Chchd10* expression (*n* = 46, β_methylation_ = −0.54, *p* = 5.36 × 10^−5^). (I) Example of a DMR changed in the hippocampus within the *Tshz3* gene at all ages (*n* = 24). (J) Added variable plot of Z‐scaled values of chr7:36723204 methylation and *Tshz3* expression (*n* = 46, β_methylation_ = −0.85, *p* = 2.08 × 10^−12^).

We applied a similar integration approach to analyze diet‐associated DEGs and DMCs. Specifically, we examined the overlap between the top 2000 choline‐protected genes identified in our complementary study (Bellio et al. 2025) (Figure 4A–C). We define choline‐protected genes in our AD model mice as those that are differentially expressed in control diet *App* ^*NL‐G‐F*^ mice versus WT, but not in PCS *App* ^*NL‐G‐F*^ mice versus PCS WT mice, indicating choline supplementation prevented genotype‐associated changes. Eighty‐three percent of the top 2000 choline‐protected genes in the cortex were differentially methylated, while 37% were differentially methylated in the hippocampus, suggesting that altered DNA methylation may regulate these genes. Additionally, we computed overlaps between DMGs and DEGs in PCS and control diet *App* ^*NL‐G‐F*^ mice at each age. Interestingly, DEGs and DMCs did not exhibit a negative correlation in either the cortex or hippocampus (Figure 4B,C).

We performed linear regression on CpG‐mRNA pairs changed by PCS. We identified 160 significantly correlated local CpG‐mRNA pairs in the cortex (Figure 4D, Supporting Information File S3) and 162 in the hippocampus (Figure 4E, Supporting Information File S3). The genes whose methylation and expression were changed by diet were preferentially related to synaptic functions based on gene set enrichment, and notable synaptic genes included *Bdnf, Gabbr2, Dlgap4*, and *Synj2* (Figure 4F). In the cortex, DMCs tended not to be present in DMRs, but rather as single cytosines, such as in *Chchd10* (Figure 4G). Only one CpG was differentially methylated due to diet in this region; however, it was strongly associated with transcription (*n* = 46, Standardized β_methylation_ = −0.54, *p* = 5.36 × 10^−5^, Figure 4H). In the hippocampus, the most prominent DMR was within *Tshz3*, a transcription factor potentially involved in APP processing (Louwersheimer et al. 2016). Methylation of CpGs in two distant parts of the gene sequence was associated with expression (Figure 4I), with the most strongly associated CpG shown (*n* = 46, Standardized β_methylation_ = −0.85, *p* = 2.08 × 10^−12^, Figure 4J).

### 2.6 Methylation of Distal Enhancers Mediates Long‐Range Regulation of Gene Expression

**FIGURE 5:**
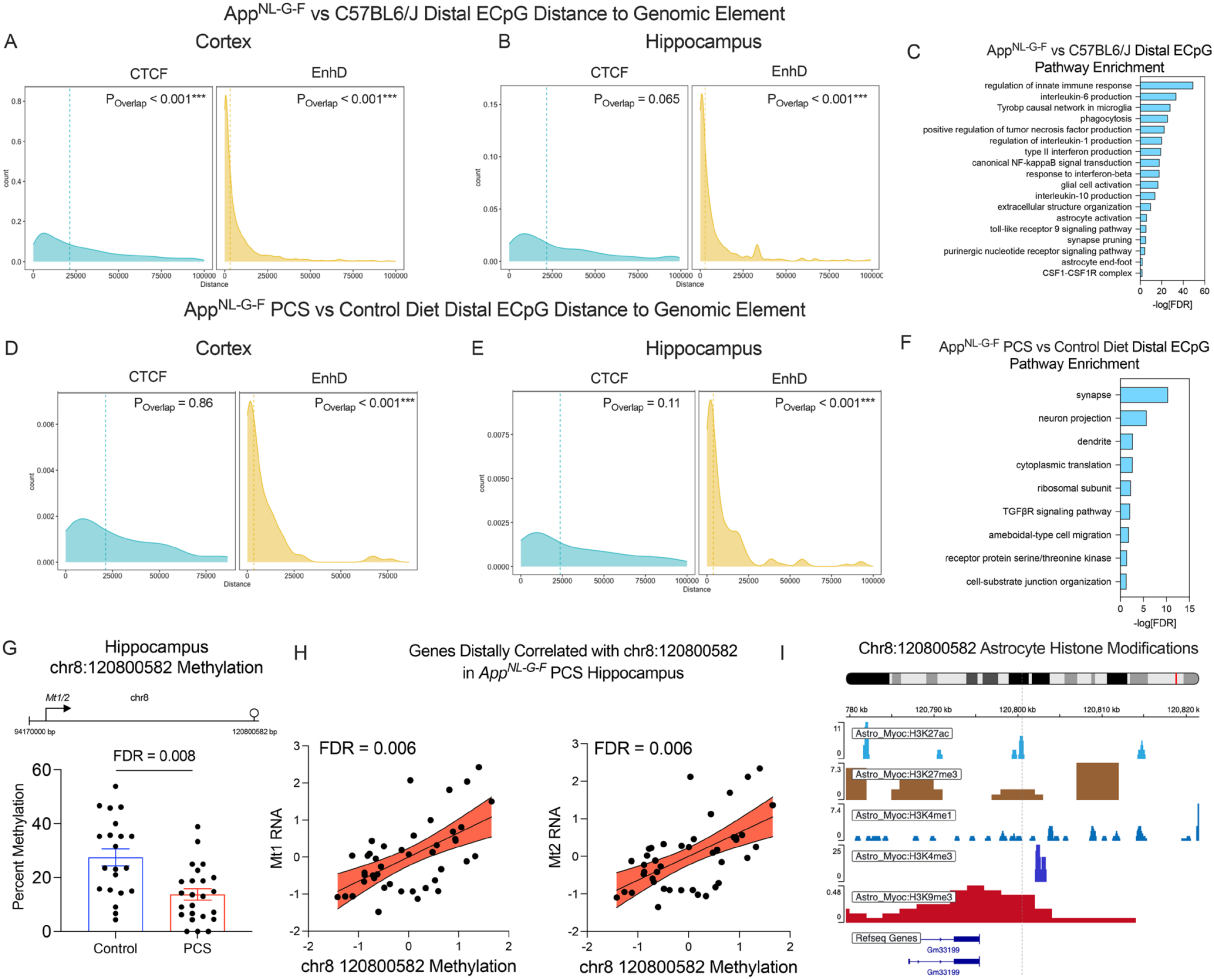
Distal enhancer methylation is associated with long‐range coordination of gene expression. Each DMC with a greater than 5% change in methylation was regressed against each DEG with at least a 0.1 log_2_ fold change on the same chromosome, controlling for age, sex, and cell proportion. Analysis was conducted between *App* ^*NL‐G‐F*^ mice vs. WT and PCS vs. control diet *App* ^*NL‐G‐F*^ mice. Distance between ECpGs and the CTCF or EnhD loci in the (A) cortex and (B) hippocampus. P values calculated by a permutation test between a ± 1 kb window around ECpGs and regulatory regions. (C) Pathway enrichment analysis of genes associated with distal CpG methylation in *App* ^*NL‐G‐F*^ mice. Distance between ECpGs and the CTCF or EnhD loci in the (D) cortex and (E) hippocampus. P values calculated by a permutation test between a ± 1 kb window around ECpGs and regulatory regions. (F) Pathway enrichment analysis of genes associated with distal CpG methylation in *App* ^*NL‐G‐F*^ PCS mice. (G‐H) RNA expression of *Mt1‐2* is distally associated with chr8:120800582 methylation in the hippocampus. (I) ChIP‐seq peaks from astrocytes show chr8:120800582 (dotted line) is located within a polycomb‐responsive element with both H3K27ac and H3K27me3 peaks.

Far‐acting genomic elements are known to interact beyond the boundaries of topologically associated domains (TADs), and these ultra‐long interacting regions are preferentially enriched for CpG islands (Friman et al. 2023). To determine potential mechanisms of methylation‐dependent gene regulation distally, we correlated DMCs with DEGs located on the same chromosome. Cis DMG‐DEG pairs, defined as within 5 kb of the transcription start site or within the gene body, were excluded from this analysis. Overall, we observed 54,330 distal CpG‐mRNA pairs in the cortex and 20,172 in the hippocampus that were significantly correlated below the FDR cutoff of 0.05 when controlling for age, sex, and cell proportion (Supporting Information File S4). One mechanism by which DNAm can regulate gene expression is through coordination with distal enhancers, as CpG islands adjacent to enhancer elements can prime their activity (Pachano et al. 2021). We calculated the distance of each distal ECpG to its nearest distal regulatory element, defined as “EnhD” (distal enhancer) or “CTCF” as CTCF regulates chromatin looping, in the ENCODE cCRE database (Moore et al. 2020). There was a statistically significant enrichment of CTCF and EnhD signatures within a 1 kb window around ECpGs, and we found that, on average, distal ECpGs were located closer to EnhD regions compared to CTCF (CTCF median distance = 28,024, EnhD median distance = 3,631 bp, Figure 5A). In the hippocampus, there was a statistically significant enrichment for EnhD within a 1 kb window around ECpGs, with EnhD signatures located closer than CTCF sites (CTCF median distance = 32,000, EnhD median distance = 3,571 bp, Figure 5B). Gene set enrichment analysis revealed that genes distally associated with altered DNAm are overwhelmingly involved in regulating inflammation in AD (Figure 5C).

As an example of distal association of DNAm with gene expression, the correlation between methylation of a 1062 bp hypermethylated DMR in the hippocampus spanning chr17:39843927‐39844989 and gene expression is shown (Figure S14A). Notably, this DMR, located in a CpG island within the Gm26917 noncoding RNA gene that is known to regulate inflammation in macrophages and microglia in mice (Weller et al. 2022; Zhao et al. 2022), is correlated with the expression of several genes involved in microglial inflammation, including *Trem2, Aif1* (encoding IBA1), *Tnf*, and complement proteins *C3* and *C4b* (Figure S14B). The association between chr17:39844008 methylation and *Aif1* expression is shown, highlighting a positive correlation between methylation of this DMR and gene expression (Figure S14C). A database of single‐cell chromatin accessibility reveals that this DMR is enriched for both the repressive H3K27me3 and permissive H3K4me1 marks in microglia, but not neurons or astrocytes (Zhu et al. 2021) (Figure S14D). Given that these marks are mutually exclusive, this suggests that this region is a distal repressor that is selectively hypermethylated in inflammatory microglial subtypes, leading to upregulated gene expression.

When assessing distal gene expression regulation due to diet‐induced methylation changes, we observed 120 significantly correlated CpG‐mRNA pairs in the cortex and 529 in the hippocampus (Supporting Information File S4). Distal CpGs in both the cortex and hippocampus were enriched for EnhD, but not CTCF sites (Cortex CTCF median distance = 25,683, EnhD median distance = 4,247 bp, Figure 5D, Hippocampus CTCF median = 29,621, EnhD median distance = 3,859 bp, Figure 5E). Gene set enrichment on genes associated with distal ECpGs revealed a role in synaptic organization, dendrite morphogenesis, and translation (Figure 5F).

One notable correlation was between chr8:120800582 and the Metallothionein (*Mt*) gene cluster. chr8:120800582 is located downstream of *Mt1* (TSS: chr8:94179245) and was hypomethylated in PCS mice (% change = 13.4%, FDR = 0.008, Figure 5G). Likewise, its methylation was positively correlated with gene expression of *Mt* genes (*Mt1: n* = 43, Standardized β_methylatio*n*_ = 0.58, FDR = 0.006, *Mt2: n* = 43, Standardized β_methylation_ = 0.58, FDR = 0.006, Figure 5H). Metallothioneins have been implicated in AD pathophysiology, and *Mt1* and *Mt2* are mainly expressed in astrocytes (de Vries et al. 2024; Juárez‐Rebollar et al. 2017). To further probe this CpG, we used previously published histone modification ChIP‐seq data to assess chromatin accessibility in astrocytes (Zhu et al. 2021). Chr8:120800582 is located within an astrocyte H3K27me3 and H3K27ac peak, indicating that it may be differentially regulated by PRC2 and is characteristic of an enhancer (Figure 5I).

### 2.7 Identification of Differentially Methylated Regions

Considering distal and local ECpGs, we identified DMRs by grouping at least 2 ECpGs within 500 bp together. We identified 763 DMRs in the cortex and 93 DMRs in the hippocampus that were associated with gene expression (Supporting Information File S5). The DMR containing the most ECpGs contained 48 hippocampal ECpGs and was located within the *Tmem267* gene (Figure S15). This DMR was also negatively correlated with *Tmem267* expression, as well as negatively correlated with the expression of microglial genes such as *Hexb* and *Cd180*, suggesting a role in inflammation. HOMER motif analysis of DMRs revealed an enrichment of ETS family transcription factors in expression‐associated DMRs (Supporting Information File S6, Figure S15). Several ETS transcription factors had differential expression in *App* ^*NL‐G‐F*^ mice in the cortex and hippocampus (Figure S15). In PCS mice, we identified fewer ECpGs located within DMRs. We identified 23 DMRs in the cortex and 12 DMRs in the hippocampus; however, these DMRs were generally short, with the longest containing 4 CpGs in the *Pcdhga* locus (Supporting Information File S5).

### 2.8 CpG Methylation Is Associated With Amyloid Plaque Burden in *App* ^*NL*^ ^*‐G‐F*^ Mice

**FIGURE 6:**
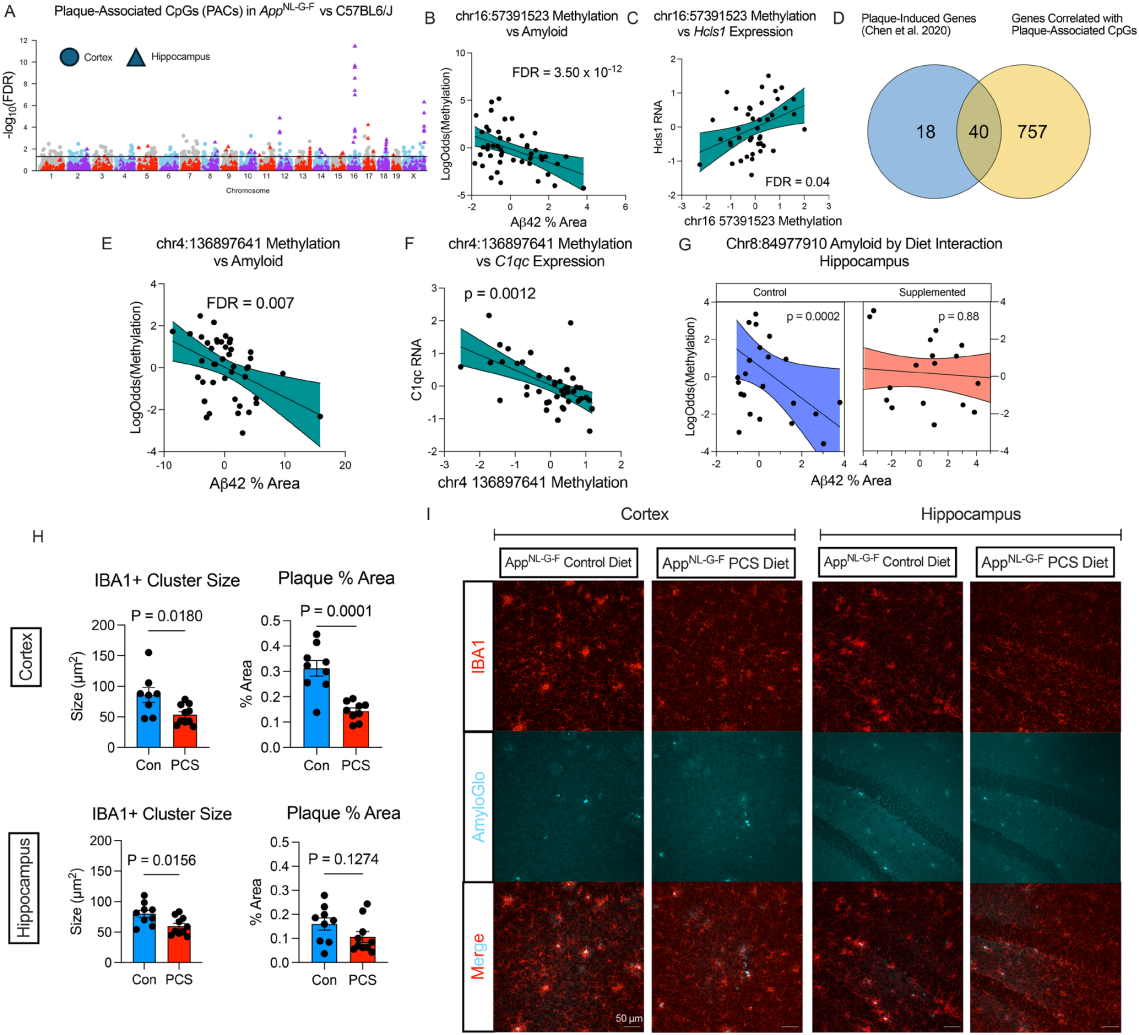
Amyloid burden is associated with DNA methylation and correlates with plaque‐induced genes. Methylation of expression CpGs was regressed against Aβ42 percent area using a general linear model with a logarithmic link, controlling for age, sex, and diet. (A) Manhattan plot of amyloid‐associated CpGs in the hippocampus and cortex (FDR cutoff 0.05). (B) Correlation of Z‐score normalized chr16:57391523 log odds methylation and percent amyloid area in the hippocampus of *App* ^*NL‐G‐F*^ mice (*n* = 47, β_amyloid_ = −0.245, FDR = 3.5 × 10^−12^). (C) Correlation of Z‐score normalized chr16:57391523 methylation and *Hcls1* RNA counts in the hippocampus by linear regression controlling for age, sex, and cell proportion (*n* = 43, β_methylation_ = 0.40, FDR = 0.04). (D) Comparison of genes correlated with plaque‐associated CpG methylation with plaque‐induced gene dataset from Chen et al. (E) Correlation of Z‐score normalized log odds methylation of chr4:136897641 and percent amyloid area in the cortex of *App* ^*NL‐G‐F*^ mice (β_amyloid_ = −0.075, FDR = 0.007). (F) Correlation of Z‐score normalized chr4:1368907641 methylation and *C1qc* RNA counts in the cortex of *App* ^*NL‐G‐F*^ and WT mice (*n* = 43, β_methylation_ = −0.38, *p* = 0.0013). (G) Regression was performed with an additional diet by amyloid interaction. Example of how perinatal choline supplementation can prevent suppression of chr8:84977910 methylation, located within *Junb*, in the hippocampus (*n* = 24, β_Control_ = −0.36, FDR = 0.0002, *n* = 23, β_Supplemented_ = 0.05, FDR = 0.88). (H) Quantification of IBA1 cell clustering and plaque area in PCS and control diet *App* ^*NL‐G‐F*^ mice hippocampus and cortex. The plaque area analyzed by Amylo‐Glo is lower than that assessed with the antibody staining in B and E, as the latter method is more sensitive to the diffuse amyloid than Amylo‐Glo that stains dense‐core fibrillar amyloid. (I) Representative 20× confocal images of staining of IBA1 and dense‐core amyloid plaques (Amylo‐Glo) in the cortex and hippocampus of PCS and control diet *App* ^*NL‐G‐F*^ mice.

We integrated our amyloid staining data with DNA methylation to identify plaque‐associated CpGs (PACs) by general linear model analysis, with Aβ42‐staining percent area as the independent variable and DNAm as the dependent variable (Bellio et al. 2024). In this analysis, we included ECpGs associated with diet or genotype in the cortex or hippocampus to relate PACs to gene expression. Within genotype‐associated ECpGs, we identified 252 amyloid‐associated CpGs in the cortex and 62 in the hippocampus (Figure 6A, Supporting Information File S7). The 8 CpGs with the strongest amyloid correlation across both regions were within a 56 bp window spanning from chr16:57391515 to 57391571 in the *Cmss1* and *Filip1l* genes. The individual CpG with the strongest association with amyloid was chr16:57391523 (*n* = 47, β_amyloid_ = −0.245, FDR = 3.5 × 10^−12^, Figure 6B). Interestingly, the methylation level of these CpGs was not correlated with the expression of *Cmss1* or *Filip1l* but rather correlated positively with the expression of a distal gene, *Hcls1*—encoding an actin‐binding protein associated with LPS‐induced microglial activation (Castro‐Ochoa et al. 2019; Guergues et al. 2020)—with the relationship between chr16:57391523 methylation and *Hcls1* expression shown as an example (*n* = 43, Standardized β_methylation_ = 0.40, FDR = 0.04, Figure 6C). Given that microglial activation is required for phagocytosis of Aβ plaques, this suggests that DNA methylation may play a role in mediating the link between amyloid sensing and transcriptional response. We compared genes correlated with PACs with the plaque‐induced gene (PIG) module described by Chen et al. (2020). We found that 40 PIGs were also associated with PAC methylation, including *Apoe, C1q, Trem2, and Tyrobp* (Figure 6D). As an example, the relationship between the Aβ plaque area, chr4:136897641 methylation, and *C1qc* expression in the cortex is shown (Figure 6E,F). Amyloid area was significantly associated with hypomethylation of chr4:136897641 (*n* = 43, β_amyloid_ = −0.075, FDR = 0.007, Figure 6E), which in turn is associated with upregulation of *C1qc* (*n* = 43, Standardized β_methylation_ = −0.38, *p* = 0.0013, Figure 6F). This suggests that DNA methylation may play a role in the regulation of PIGs.

Our previous work has demonstrated that PCS reduced amyloidosis in *App* ^*NL‐G‐F*^ mice (Bellio et al. 2024). Therefore, we stratified mice by diet and tested each diet‐associated ECpG for its association with amyloid. We identified 20 ECpGs in the cortex and 82 ECpGs in the hippocampus whose methylation level was significantly correlated with amyloid in one diet group, but not the other (Supporting Information File S8). One instance was with the CpG chr8:84977910 located within the AP‐1 component *Junb* (Control diet FDR = 0.0002, PCR FDR = 0.88, Figure 6G). Methylation of this CpG was positively correlated with *Junb* expression, and increased amyloid correlated with decreased DNAm in control diet *App* ^*NL‐G‐F*^ mice (*n* = 24, β_Control_ = −0.36, FDR = 0.0002). However, in PCS *App* ^*NL‐G‐F*^ mice, amyloid was not associated with decreased DNAm (*n* = 23, β_Supplemented_ = −0.05, FDR = 0.88), suggesting PCS can prevent amyloid‐induced alteration of DNAm. As microglia are the primary immune cells responding to amyloidosis, we performed immunofluorescence staining for the microglial marker IBA1 in the sections of the somatosensory cortex and dentate gyrus of the hippocampus in 12‐month‐old *App* ^*NL‐G‐F*^ mice fed a control or PCS diet to assess microglial reaction to plaques as a correlate linking altered DNAm in response to plaque area. Twelve months was chosen as this age represents an advanced disease state. We co‐stained these sections for dense core plaques using Amylo‐Glo to assess microglial engagement of plaques. Microglia demonstrated reduced clustering in the cortex and hippocampus, and we observed fewer plaques in the cortex of PCS mice, consistent with previous studies (Figure 6H,I, Bellio et al. 2024). We also found that, in both the cortex and hippocampus of PCS mice, microglia tended to possess homeostatic morphology, with fewer ameboid microglia (Figure 6H,I). Overall, these data indicate that PCS reduces the number of inflammatory plaque‐associated microglia, providing a potential mechanism linking Aβ42 area and DNAm.

## 3 Discussion

The data presented here show that the *App* ^*NL‐G‐F*^ AD model mice are characterized by age‐dependent abnormalities in the cerebral cortical and hippocampal DNA methylome, and that some of these abnormalities are ameliorated by PCS. Age is the strongest risk factor for AD, and the recognition of the robust and reproducible age‐related associations of changes in DNAm of specific CpGs and genomic regions in multiple species has inspired the construction of DNAm epigenetic clocks that link tissue‐specific DNAm patterns to the individuals' chronological age (Moqri et al. 2024). Previous analyses of postmortem brain samples from several human cohorts suggest that AD is characterized by acceleration of these clocks (i.e., AD subjects appear older than their age) (Levine et al. 2015; Sommerer et al. 2023). Using a high‐coverage version of RRBS and a DMR‐based algorithm to generate murine epigenetic clocks, Coninx et al. reported that 3xTg AD model mice are characterized by a marked DNAm clock acceleration in the cerebral cortex and, to a lesser degree, in the hippocampus (Coninx et al. 2020). Although our RRBS sequencing depth was insufficient to generate a clock (Simpson et al. 2023), our data show dynamic differential methylation of all the clock DMRs employed by Coninx et al. (e.g., *Ntng2*, *Dleu2*, *Rapgefl1*, *Dapk1*, *Exoc3l2*, and *Tshz3*) in association with *App* ^*NL‐G‐F*^ genotype and PCS, particularly in the cortex. [UCSC Genome Browser custom tracks showing all the DMCs in our data can be found here: https://genome.ucsc.edu/s/krunican/mm10_AppNLGF_DMCs].

The age‐dependent abnormalities of DNA methylation in *App* ^*NL‐G‐F*^ mice follow the time course of brain amyloid plaque accumulation, which triples between 3 and 9 months of age (Bellio et al. 2024). Similarly, the number of DMGs more than doubled over this period in both cortex and hippocampus. In the cortex, half of the DMGs observed at 3 months of age remain abnormally methylated until 12 months. These data indicate that abnormal DNAm is an early, persistent, and progressive response to the amyloidosis and/or the amyloid‐evoked neuroinflammation that characterizes this AD model (Chen et al. 2020). Although the number of DMGs was approximately tenfold higher in the cortex as compared to the hippocampus, up to 80% of the hippocampal DMGs were also abnormally methylated in the cortex, suggesting that there exists a core set of loci whose methylation is altered in response to the amyloid accumulation in these mice. This brain region dependence of abnormal DNAm patterns in this model may be related to the progression of amyloidosis, which in *App* ^*NL‐G‐F*^ mice begins in the cortex and spreads to the hippocampus in more advanced disease. Furthermore, it has been shown that there are spatial differences in average methylation between regions in the healthy adult mouse brain, which may suggest different susceptibility to DNAm changes in the context of disease or choline supplementation (Liu et al. 2021).

We have demonstrated that CpG methylation is associated with the expression of genes related to microglial activation and inflammation in *App* ^*NL‐G‐F*^ mice, including the *C1q* locus. C1q is known to bind amyloid plaques and facilitate their phagocytosis (Webster et al. 2000; Zanjani et al. 2005). However, it is also involved in synapse elimination and has been implicated in synapse loss and neuropathology in AD mouse models (Dejanovic et al. 2022; Fonseca et al. 2004; Hong et al. 2016). DNAm has been implicated in the repression of *C1q* in peripheral monocytes, and our data suggest that DNAm regulates *C1q* expression in AD. Moreover, our expression DMR motif enrichment is consistent with previous findings that implicate the inflammation‐associated ETS‐family transcription factors in AD development (Gjoneska et al. 2015). ETS1 protein expression is reportedly upregulated in postmortem AD brains and co‐localizes with microglia and vasculature (Jantaratnotai et al. 2013). ETS2 regulates macrophage inflammatory states in the periphery, suggesting related transcription factors may play a similar role in microglia (Stankey et al. 2024).

We have shown that PCS‐associated changes in DNAm are associated with the expression of genes in synaptic function, mitochondrial activity, and protein translation. The gene most strongly associated with DNAm in PCS was *Tshz3*, an autism‐related transcription factor that shows promoter hypomethylation in PCS. Loss of *Tshz3* in cortical projection neurons is associated with autism phenotypes (Caubit et al. 2022). Expression of *Tshz3* is decreased in *App* ^*NL‐G‐F*^ mice and increased by PCS in the hippocampus, possibly through altering DNAm. Interestingly, the Drosophila analogue *tsh* can prevent pathologic amyloid processing, suggesting *Tshz3* could be a potential target for therapeutics (Moran et al. 2013). Additional notable loci include *Chchd10*, a mitochondrial gene implicated in neurodegeneration (Ikeda et al. 2022) and a promoter region of brain‐derived neurotrophic factor, *Bdnf*. The latter is highly conserved among mammals and includes a common protein‐coding exon and several upstream promoters whose methylation is dynamic and associated with neurological and psychiatric diseases (Treble‐Barna et al. 2023). Most notably, alternative methylation of *Bdnf* is associated with mRNA expression of specific transcript variants, highlighting the dynamic role of DNAm in modulating transcript usage (Lubin et al. 2008). In PCS mice, decreased DNAm in the region identified in Figure 2 is associated with increased BDNF levels, highlighting how hypomethylation of certain loci can be neuroprotective.

Specifically, we demonstrated that Aβ42 plaque load correlates with DNAm of certain CpGs, which we dubbed PACs. One possible mechanism of how Aβ affects methylation is through the inhibition of the excitatory amino acid transporter 3 (EAAT3), which is partially responsible for the impaired glutamate uptake and neuronal hyperexcitability observed in AD models (Findley et al. 2024). Because EAAT3 is also a transporter of cysteine, its inhibition by Aβ may lead, indirectly, to reduced availability of AdoMet (Hodgson et al. 2013). Previous studies of astrocytes in traumatic brain injury have shown spatially restricted changes in DNAm near the injury site, implicating it in the local response to brain insult (Cuautle et al. 2024). Through our integration with RNA‐seq data, we were able to assess the potential role of PACs in gene expression. Certain PACs, such as chr4:136897641, show a correlation between *C1q* RNA expression and Aβ plaque load, suggesting that Aβ causes CpG hypomethylation to induce gene expression. This is consistent with a study of Chen et al., where *C1qc* and several other genes were designated as plaque‐induced genes (PIGs) using spatial transcriptomics in *App* ^*NL‐G‐F*^ mice (Chen et al. 2020). Our data suggest that changes in DNAm may be an intermediate step in linking amyloid burden to PIG expression. However, many PACs showed the opposite direction of correlation between amyloid‐CpGs and CpGs‐genes, suggesting gene silencing due to Aβ. For instance, methylation of the CpG island located from chr16:57391515 to 57391571 was negatively correlated with amyloid burden but positively correlated with gene expression of *Hcls1*. *Hcls1* has been previously linked to AD and the relationship between amyloid and tau in the hippocampus of ADLP^APT^ mice (Kim et al. 2018). Surprisingly, *Hcls1* expression in this model peaked in early disease and decreased at later time points. As plaque density increases with age, leading to decreased methylation and decreased expression, our model of PACs can provide an explanation for this temporal expression pattern. It should be noted that the causal order of events in the relationship between DNAm, Aβ accumulation, and RNA expression cannot be conclusively determined, as our analysis is correlational.

We identified multiple CpGs in *App* ^*NL‐G‐F*^ mice that differ in their response to amyloid due to PCS. Specific examples of PCS‐modulated PACs are located near *Psd4, Cdh18*, and *Slc17a6*, encoding synaptic and neuronal proteins. These findings are consistent with our previous studies showing that PCS was protective against cognitive defects in this animal cohort (Bellio et al. 2024) and studies linking synaptic integrity and gene expression with resilience to AD pathology in humans (Neuner et al. 2022; Perez‐Nievas et al. 2013; Yu et al. 2020). Additionally, PCS has been shown to lower brain homocysteine levels in AD mouse models, which may directly alter DNAm in PACs by preventing Aβ‐associated changes (Velazquez et al. 2020).

This study provided an opportunity to assess correlations between the methylation of local and distal CpGs and gene expression in *App* ^*NL‐G‐F*^ mice. A recent paper by Liu et al. demonstrated that 73% of the links between differentially methylated regions and gene expression are distal, highlighting the role of CpG methylation outside of promoter regions (Liu et al. 2023). Additionally, Kim et al. showed that distal DNAm upwards of 10 Mb from the transcription start site is associated with gene expression. Our results show a similar distribution, where a greater number of distal rather than local CpG‐gene pairs are distally, rather than locally, correlated with gene expression (S. Kim et al. 2020). Many distal ECpGs are preferentially located near genomic regions exhibiting distal enhancer signatures, suggesting they may regulate enhancer function. Additionally, CpGs such as chr8:120800582 and DMRs such as chr17:39843927‐39844989 are located within regions harboring permissive and repressive histone marks, further suggesting that these are active regulatory regions, highlighting the potential interplay between DNAm and chromatin state. [UCSC Genome Browser custom tracks showing all expression‐linked CpGs in our data can be found here: https://genome.ucsc.edu/s/krunican/mm10%20AppNLGF%20ECpGs].

While RRBS provides a high‐resolution technique to map modified cytosines at the 5 position, particularly in CG‐rich genomic regions (e.g., CpG islands), it cannot distinguish between 5mC and 5‐hydroxymethylcytosine (5hmC). This lack of specificity represents a limitation of our study, as 5hmC is not only an intermediate in DNA demethylation but also serves as a *bona fide* epigenomic mark modulating transcription and RNA splicing during aging (Occean et al. 2024). However, the age‐related 5hmC changes are found mostly in regions distal from CpG islands and in gene bodies, including in human autopsy cortical samples (Zhao et al. 2025), suggesting that the vast majority of our 5mC data are valid.

Overall, we have demonstrated that *App* ^*NL‐G‐F*^ genotype and PCS induce age‐ and brain‐region‐dependent DNA methylation changes that correlate with amyloid burden. Moreover, we have shown that DMCs are locally and distally associated with gene expression, suggesting a role of DNAm in regulating transcriptomic differences due to *App* ^*NL‐G‐F*^ genotype and perinatal diet. Our studies delineate an epigenetic mechanism underpinning the effect of PCS in an AD model and suggest that targeting the DNA methylome, using pharmacologic or nutritional approaches, as described here, may represent a promising future direction for AD prevention and/or therapy.

## 4 Methods

### 4.1 Animals

Animals were maintained as previously described (Bellio et al. 2024). C57BL/6J wild type (WT, Charles River Laboratory) and *App* ^*NL‐G‐F*^ mice (RIKEN, Japan) were kept on a 12‐h reverse light cycle (11 pm–11 am lights on). Mice were fed the AIN76A diet (66% carbohydrates, 20.3% proteins, 5% fat) containing either 1.1 g/kg (control, Dyets Inc. #110098) or 5 g/kg (supplemented, Dyets Inc. #110184) choline chloride. From one week prior to mating to postnatal day 21, mothers were fed either a control or a supplemented diet. Following weaning, all offspring, regardless of maternal diet, were fed the control diet *ad libitum*. Mice were euthanized at 3, 6, 9, and 12 months by CO_2_ inhalation. All mice were subjected to behavioral testing (open field, elevated plus maze, Barnes maze, and contextual fear conditioning testing) prior to euthanasia as previously described (Bellio et al. 2024). A total of 96 mice were included in this study (*n* = 3 per age/genotype/diet/sex). The full study design is included in Figure S16.

### 4.2 Tissue and DNA/RNA Extraction

Following euthanasia, mice were decapitated, and their brains were immediately removed. The left hemisphere was quickly placed in Leibovitz L15 media (Gibco #41300039) on ice, and the hippocampus and cortex were isolated. Cortical regions were consistent between mice and encompassed a piece containing motor, cingulate, and somatosensory cortex. Tissue was frozen on dry ice and kept at −80°C. Genomic DNA and RNA were isolated from mouse hippocampus or cortex using the Quick DNA/RNA MiniPrep Plus Kit (Cat. D7003, Zymo). The right hemisphere was fixed in 8 mL periodate‐lysine‐paraformaldehyde (PLP, 10 mM sodium periodate, 4% paraformaldehyde, 75 mM lysine, pH 7.4) at 4°C for 24 h. Brains were cryoprotected for 24 h each in 10%, then 20% glycerol in 2% DMSO/0.1 M phosphate buffer, pH 7.3.

### 4.3 Reduced Representation Bisulfite Sequencing

DNA was shipped to Zymo Research (Irvine, CA) for RRBS analysis. Briefly, 30 ng of input DNA was digested with Mspl, and RRBS libraries were generated using 5‐mC adapters. Samples were bisulfite‐treated (EZ DNA Methylation‐Lightning Kit, Cat. D4003, Zymo) and sequenced on the Illumina NovaSeq X with 150‐bp paired‐end reads. Reads were trimmed using Trim Galore, and FASTQ quality control was conducted using FastQC. Genome alignment was performed using Bismark, and methylation proportions for each CpG were calculated using MethylDackel. BedGraph files were generated and read into R for downstream analysis.

### 4.4 Methylome Deconvolution and Cell Proportion Estimation

Cell proportions were deconvoluted using RRBS data with the DecompPipeline R package (Lutsik et al. 2017; Scherer et al. 2020). This pipeline identifies latent methylation components (LMCs) that roughly correspond to the cell type proportion in a sample. For deconvolution, CpGs containing fewer than 3 reads in all samples, within the 99th percentile of coverage, on sex chromosomes, or missing reads were filtered for quality control. The 5000 most variable CpG sites based on all samples in the hippocampus or cortex were selected for use in DecompPipeline. DecompPipeline was run with 10 initiations, 10‐fold validation, and 300 maximum iterations. A lambda value of 10^−3^ for both tissues was used, while 3 and 6 components were used in the cortex and hippocampus, respectively. To assign identities to LMCs, we correlated per‐sample LMC values with RNA counts. We identified 2 latent methylation components (LMCs) in each brain region that roughly correspond to the relative proportions of neurons (LMC2 in the cortex and hippocampus, based on correlation with neuronal markers) and microglia (LMC3 in the cortex and LMC6 in the hippocampus, based on correlation with microglial markers) (Figure S2). These components were included in downstream regression analysis to control for variations in cell proportion.

### 4.5 Differential Methylation Analysis

Differentially‐methylated CpG sites (DMCs) were called using the R package methylKit (Akalin et al. 2012). CpGs were filtered if they had a read depth less than 10 or were within the 99.9th percentile of reads, indicating PCR bias. Additionally, a CpG was required to be covered in at least 50% of samples to be included in downstream analysis. A CpG was considered differentially methylated if it had a sliding linear model‐corrected *p*‐value < 0.05. CpGs were annotated to the GRCm38 reference genome using the annotatr R package. A CpG was mapped to a gene if it was within the transcriptional start site (TSS), intron, exon, 5′ and 3′ untranslated regions (UTR), promoter (1 kb upstream), or within 1 to 5 kb upstream of the TSS. Differential methylation analysis was broadly conducted using the following regression formula:

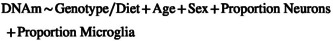

With genotype representing *App* ^*NL‐G‐F*^ control diet versus WT control diet animals, and diet representing *App* ^*NL‐G‐F*^ supplemented diet versus *App* ^*NL‐G‐F*^ control diet. Age was not included as a covariate when stratifying by age.

### 4.6 RNA Sequencing

Details about the RNA‐seq pipeline can be found in our companion study (Bellio et al. 2025). Briefly, RNA sequencing was conducted by MedGenome Inc. (Foster City, CA), and library preparation was conducted with the Takara SMARTer Stranded Total RNA‐Seq Kit v2—Pico Input. Sequencing was done on an Illumina NovaSeq 6000 with 20 million paired‐end reads of 100 bp. FASTQ files were trimmed using trimmomatic, aligned to the GRCm39 transcriptome, and quantified using SALMON (Bolger et al. 2014; Patro et al. 2017). Differentially expressed genes (DEGs) were determined using DESeq2, with independent hypothesis weighting performed post hoc to maximize power (Love et al. 2014). DEGs were calculated at 3, 6, 9, and 12 months, as well as all ages together, controlling for age. To investigate the effect of genotype, DEGs between *App* ^*NL‐G‐F*^ and WT at a Benjamini–Hochberg FDR < 0.05 were included for downstream analysis. DEGs between *App* ^*NL‐G‐F*^ supplemented and *App* ^*NL‐G‐F*^ control diet animals were required to meet a relaxed FDR < 0.1 cutoff. Additionally, we determined choline‐protected genes by intersecting DEGs between the control diet *App* ^*NL‐G‐F*^ and WT (FDR < 0.05) that are no longer significantly changed in the supplemented diet *App* ^*NL‐G‐F*^ and WT (FDR > 0.05), indicating choline prevented a change in expression between WT and *App* ^*NL‐G‐F*^. Log2FoldChange for choline‐protected genes was determined by the difference in Log2FoldChange between supplemented and control diets. The top 2000 choline‐protected genes based on FDR between the control diet *App* ^*NL‐G‐F*^ and WT were included for downstream analysis.

### 4.7 DNA Methylation‐RNA Sequencing Integration

To identify CpGs linked to gene expression locally, we performed multiple linear regression between the DESeq2 variance‐stabilizing transformed counts for a gene and the percent methylation of a CpG mapped to the same gene using the following formula:

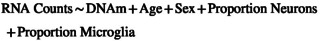

CpGs located within genes that showed a percent change > 2.5% were included for downstream analysis. Genes with a log2 fold change > 0.1 were included for analysis. This cutoff was chosen to filter genes that had a statistically significant change due to low counts, which can result in inflated *p*‐values and show low fold changes when the DESeq2 fold change shrinkage algorithm is run. The hippocampus and cortex were each tested separately. CpG methylation level—mRNA abundance pairs with a multiple linear regression *p*‐value for methylation < 0.05 were termed “expression CpGs” and were investigated further. Expression CpGs from the hippocampus and cortex were pooled, and pathway analysis was conducted using g:Profiler (Reimand et al. 2007).

To determine the effects of DNA methylation distally, we performed multiple linear regression between DMCs and DEGs located within the same chromosome using the same formula previously mentioned. Due to the large number of intergenic DMCs and to reduce multiple testing, we used a stricter 5% change in methylation cutoff for distal DMC‐gene correlation. Due to the extensive transcriptomic changes and to minimize multiple testing in the *App* ^*NL‐G‐F*^ versus WT group, the top 1000 DEGs by FDR were selected. Sex, age, and cell proportion (via LMCs) were controlled for in regression. CpG‐mRNA pairs with a Benjamini–Hochberg adjusted FDR < 0.05 were considered significant. Overlap of ECpGs with genomic elements (EnhD and CTCF) was done using the regioneR package (Gel et al. 2016).

Differentially methylated regions (DMRs) were generated by clustering individual CpGs within 500 bp of each other. At least two CpGs were required to form a region. DMR motif enrichment was performed using the HOMER motif enrichment tool with a region size of 200 (Heinz et al. 2010).

### 4.8 Integration of Amyloid Burden and DNAm

Anterior hippocampus sections containing the hippocampus and primary somatosensory cortex were washed with PBS for 10 min and transferred to 70% formic acid for 1 min. Tissue was blocked in 10% goat serum in 0.1 M PBS for 1 h and incubated with anti‐Aβ42 rabbit antibody at 1:1000 (Invitrogen #700254) in 0.3% Triton‐X 100, 2% goat serum, and 0.008% sodium azide overnight. Sections were washed in 0.1 M PBS 3 times for 10 min and were incubated with goat anti‐rabbit IgG (H + L) HRP conjugated antibody at 1:750 (Millipore #AP307P) in 2% goat serum in 0.1 M PBS for 3 h at room temperature with agitation. Tissue sections were washed 3 times for 10 min in 0.1 M PBS as before and were developed in a diaminobenzidine, sodium imidazole, and hydrogen peroxide solution. Mounted slides were imaged on an Olympus B061 microscope at a 2× objective, capturing the whole hippocampus and cortex. The hippocampus and cortex were outlined in ImageJ, and the Aβ42‐positive area was quantified in ImageJ. Generalized linear models were used to correlate DNAm and percent area of Aβ42 to determine the relationship between amyloid load and methylation.

### 4.9 Determination of Tissue Amyloidosis and Microgliosis by Confocal Microscopy

Free‐floating 40 μm thick tissue sections from 12‐month‐old *App* ^*NL‐G‐F*^ mice were blocked for 1 h in 3% BSA/0.3% Triton X‐100 in PBS, followed by 15 min in 3% hydrogen peroxide/50% methanol. Microglia were stained using a primary antibody against IBA1 (Wako, 01–1974) diluted 1:1000 in blocking buffer and incubated overnight. Subsequently, sections were washed 3 times in PBS and incubated in a secondary antibody for 3 h (Donkey anti‐Rabbit AlexaFluor 594, 1:2000). Amyloid plaques were stained via Amylo‐Glo (1:100, TR‐300‐AG) and mounted using VectaShield. Images were acquired using a Zeiss 710 confocal microscope with a 20× objective lens and 1.5 μm Z‐stack optical sections. Images were analyzed in ImageJ. To quantify beta amyloidosis and IBA1 staining, regions of interest underwent maximum intensity Z‐projection, followed by automatic thresholding to reduce experimenter bias. The “Analyze Particle” function in ImageJ was used to quantify IBA1+ cluster size.

### 4.10 Statistical Methods

Statistical tests were used as mentioned. DNAm‐RNA integration was performed using multiple linear regression and partial correlation with RNA as the dependent variable. The predictors used in the regression model were CpG methylation, age, sex, LMC2/3 (Cortex only), and LMC2/6 (Hippocampus only). Data points with a Cook's distance greater than 4/*n* and a standardized residual greater than 3 were considered outliers and excluded from the analysis. Pearson's partial correlation analysis was performed controlling for age, sex, and LMCs, using the method previously stated. For distal correlation, *p*‐values were corrected using the Benjamini–Hochberg procedure. To determine the relationship between DNAm and amyloid burden, logistic regression was conducted with DNAm as the dependent variable, and Aβ42‐positive area, age, sex, and diet were included as predictors in the model. DNAm was modeled using a binomial distribution with the binomial probability *P* equal to the methylation proportion of CpG j in each sample, and prior weights set to the number of reads on CpG j. A logarithmic link function was used for regression. The Benjamini–Hochberg adjustment was performed to control the false discovery rate.

All statistical tests were carried out using R and are used as described. Plots were generated using GraphPad Prism 10 and R. Heatmaps were generated using the complexHeatmap R package (Gu et al. 2016). Circos plots were generated using the circlize R package (Gu et al. 2014).

## Author Contributions

Concept and design: J.K.B., T.J.M. Acquisition of data: T.A.B., A.K., B.Z.C., T.J.M., J.K.B. Data analyses: T.A.B., A.K., T.J.M., J.K.B., H.L., A.L. Interpretation of data: T.A.B., A.K., T.J.M., J.K.B., H.L., A.L., T.D.S. Drafting of the manuscript: T.A.B., A.K., J.K.B. Reviewing and editing: T.A.B., A.K., T.J.M., J.K.B., H.L., A.L., T.D.S.

## Conflicts of Interest

The authors declare no conflicts of interest.

## Supporting information


**Figure S1:** Annotations of DMCs changed in *AppNL‐G‐F* mice versus WT. DMCs from each age point and all ages combined were pooled for downstream annotation. (A) Genomic context annotations for DMCs in the cortex and hippocampus shows primarily promoter enrichment. (B) Distance to nearest transcription start site (TSS) for DMCs in the cortex and hippocampus. (C) Distribution of DMCs over time in the cortex colored by direction of change. (D) Distribution of DMCs over time in the hippocampus colored by direction of change. Depth in Circos plot represents density of DMCs in a region.
**Figure S2:** MeDeCom/DecompPipeline cell type deconvolution can identify major cell populations. Heatmaps showing hierarchical clustering of samples based on cell type proportion in (A) cortex and (B) hippocampus reveals separation between genotypes (n = 96). Comparison between genotypes in the (C) cortex and (D) hippocampus shows cell proportions changed in *App* ^*NL‐G‐F*^ mice. (E) LMC2 in the cortex correlates with the RNA expression of neuronal gene *Glra2* (Spearman‘s *ρ* = 0.822, *p* < 0.0001). (F) LMC3 in the cortex correlates with the RNA expression of microglial gene *Cd74 (*Spearman‘s *ρ* = 0.657, *p* < 0.0001). (G) LMC2 in the hippocampus correlates with the RNA expression of neuronal immediate early gene *Egr3 (*Spearman‘s *ρ* = 0.67, *p* < 0.0001). (H) LMC6 in the hippocampus correlates with the expression of glial protein *C3 (*Spearman‘s *ρ* = 0.569, *p* < 0.0001). Data shown as mean ± SEM.
**Figure S3:** Coverage is consistent between brain region batches in *AppNL‐G‐F* mice versus WT. (A–E) Coverage between all CpGs with a 10‐fold read depth in at least 50% of samples at each age between brain regions in *App* ^*NL‐G‐F*^ mice versus WT.
**Figure S4:** Overlap between differentially methylated cytosines and total coverage by age in *AppNL‐G‐F* mice versus WT Overlap between DMCs in the (A) cortex and (B) hippocampus. Comparison of coverage at 10‐fold read depth in at least 50% of samples for DMCs changed in *App* ^*NL‐G‐F*^ mice versus WT in the (C) cortex and (D) hippocampus.
**Figure S5:** acel70241‐sup‐0001‐FigureS1‐S16.pdf. *AppNL‐G‐F* mice show differential methylation of imprinted genes compared to WT. DMCs at each age were mapped to their nearest gene as described in the methods. Differential methylation of imprinted genes shown in the (A) cortex and (B) hippocampus. Blue represents differentially methylated in *App* ^*NL‐G‐F*^ mice compared to WT. C‐D) Heatmap showcasing RNA expression log2 fold changes denoted with the color scale and adjusted p values for imprinted genes in the cortex and hippocampus. *p < 0.05, **p < 0.01, ***p < 0.001, ****p < 0.0001.
**Figure S6:** Whole genome DMCs in *AppNL‐G‐F* mice versus WT stratified by sex. Circos plots showing genome‐wide distribution of DMCs in (A) male and (B) female cortex. (C) Quantification of DMCs over time in male and female cortex. Circos plots showing genome‐wide distribution of DMCs in (D) male and (E) female hippocampus. (F) Quantification of DMCs over time in male and female hippocampus.
**Figure S7:** acel70241‐sup‐0001‐FigureS1‐S16.pdf. *AppNL‐G‐F* mice show sex dependent X chromosome methylation compared to wild type. Reduced representation bisulfite sequencing was performed on the hippocampus and cortex of male or female *App* ^*NL‐G‐F*^ or C57BL/6J mice, controlling for age and diet (*n* = 24 per sex per genotype, *n* = 48 total). CpGs with < 0.05 *q*‐value were considered differentially methylated. X chromosome diagrams showing DMC percent change in *App* ^*NL‐G‐F*^ vs WT mice in the cortex by age in (A) females and (B) males. (C) Diagrams showing DMC percent change in *App* ^*NL‐G‐F*^ vs WT mice in the hippocampus by age in (C) females and (D) males. (E) ChrX:159627109 (located within the *Sh3kbp1* gene) is hypomethylated in male *App* ^*NL‐G‐F*^ mice at 3 months (Welch‐corrected *t* = 2.947, *p* = 0.032). ChrX:159627109 methylation does not change at 3 months in the cortex of female *App* ^*NL‐G‐F*^ mice (Welch‐corrected *t* = 1.276, *p* = 0.2331). Data shown as mean ± SEM. (F) ChrX:60893145 (located within the *Sox3* gene) is hypomethylated in female *App* ^*NL‐G‐F*^ mice at 9 months (Welch‐corrected *t* = 2.743, *p* = 0.0215). ChrX:60893145 methylation does not change at 9 months in the cortex of male *App* ^*NL‐G‐F*^ mice (Welch‐corrected *t* = 0.2194, *p* = 0.8314).
**Figure S8:** Annotations of DMCs changed in *AppNL‐G‐F* PCS versus control diet mice. DMCs from each age point and all ages combined were pooled for downstream annotation. (A) Genomic context annotations for DMCs in the cortex and hippocampus shows primarily promoter enrichment. (B) Distance to nearest transcription start site (TSS) for DMCs in the cortex and hippocampus. (C) Distribution of DMCs over time in the cortex colored by direction of change. (D) Distribution of DMCs over time in the hippocampus colored by direction of change. Depth in Circos plot represents density of DMCs in a region.
**Figure S9:** *AppNL‐G‐F* and WT PCS mice have distinct methylation changes. Circos plots showing genome‐wide distribution of DMCs in PCS versus control diet WT mice in (A) cortex and (B) hippocampus. Overlap of DMCs between *App* ^*NL‐G‐F*^ PCS vs control diet.
**Figure S10:** Coverage is consistent between brain region batches in *AppNL‐G‐F* PCS vs control diet mice. (A–E) Coverage between all CpGs with a 10‐fold read depth in at least 50% of samples at each age between brain regions in *App* ^*NL‐G‐F*^ PCS vs control diet mice.
**Figure S11:** Overlap between differentially methylated cytosines and total coverage by age in *AppNL‐G‐F* PCS vs control diet mice. Overlap between DMCs in the (A) cortex and (B) hippocampus. Comparison of coverage at 10‐fold read depth in at least 50% of samples for DMCs changed in *App* ^*NL‐G‐F*^ PCS versus control diet mice in the (C) cortex and (D) hippocampus.
**Figure S12:** *AppNL‐G‐F* mice fed a PCS diet show differential DNA methylation of imprinted genes compared to control diet. DMCs at each age were mapped to their nearest gene as described in the methods. Differential methylation of imprinted genes shown in the (A) cortex and (B) hippocampus. Blue represents differentially methylated in PCS *App* ^*NL‐G‐F*^ mice compared to control diet. (C–D) Heatmap showcasing RNA expression log2 fold changes denoted by the color scale and adjusted p values for imprinted genes in control diet *App* ^*NL‐G‐F*^ vs WT and PCS diet *App* ^*NL‐G‐F*^ vs WT the cortex and hippocampus. *p < 0.05, **p < 0.01, ***p < 0.001, ****p < 0.0001.
**Figure S13:** Perinatal choline supplementation can reverse genotype‐induced DNA methylation changes in *AppNL‐G‐F* mice. DMCs due to genotype (*App* ^*NL‐G‐F*^ vs WT, control diet) and diet (*App* ^*NL‐G‐F*^ PCS vs control diet) at all ages, controlling for age were compared. Number of overlapping DMCs due to genotype and diet in (A) cortex and (B) hippocampus. DMCs required to have *q* < 0.05 in both treatment groups. Heatmaps showing reversal of genotype‐dependent methylation by diet in (C) cortex and (D) hippocampus. DMGs were determined by mapping each CpG to their nearest gene according to parameters previously discussed. Overlap between DMGs in the (E) cortex and (F) hippocampus. Percent change for each DMG was calculated by averaging the percent change for each DMC. Heatmaps showing reversal of genotype‐dependent net gene methylation by diet in (G) cortex and (H) hippocampus. (I) Example of how diet can reverse genotype dependent methylation of chr8:46540075, located upstream of *Ascl1*, in the cortex at 9 months. There is a decrease in methylation in *App* ^*NL‐G‐F*^ mice on the control diet (Holm‐Sidak adjusted *p* = 0.0343), with PCS reversing genotype changes (Holm‐Sidak adjusted *p* = 0.0085). (J) Example of how diet can reverse genotype dependent methylation of chr10:94052312, located near *Fgd6*, in the hippocampus. Hypomethylation due to *App* ^*NL‐G‐F*^ genotype was reversed by PCS.
**Figure S14:** Distal DMR Associated with AD Genotype is Enriched for Repressive Histone Marks (A) Example of a DMR that is distally correlated with the expression of microglial inflammation genes (B) Magnification of region containing *C4b, Aif1, Tnf, Tap1*, and *Psmd9* (C) Association between *C4b* mRNA and methylation of chr17 39844008, located within a distal DMR (D) Diagram generated by CATlas (http://catlas.org/catlas_hub/) using data from Zhu et al. 2021. Peak enrichment of histone marks H3K27ac, H3K27me3, and H3K4me1 are shown in hippocampal CA1 neurons, astrocytes, and microglia.
**Figure S15:** Expression CpGs in *AppNL‐G‐F* mice are located within differentially methylated regions. DMRs were generated as described in the methods. (A) Representative 634 bp DMR within *Tmem267* differentially methylated in *App* ^*NL‐G‐F*^ mice and is correlated with *Tmem267* expression. (B) HOMER enrichment of DMRs in *App* ^*NL‐G‐F*^ mice reveal ETS family transcription factor sequences over‐represented in DMRs. RNA expression of differentially expressed ETS transcription factors in *App* ^*NL‐G‐F*^ mice (C) cortex and (D) hippocampus.
**Figure S16:** Study Design. Seven days prior to mating, female mice were placed on either a choline supplemented or standard diet and maintained until postnatal day 21, when pups were weaned and placed on the control diet. At 3‐, 6‐, 9‐, and 12‐months mice were sacrificed and RNA and DNA was extracted from the cortex and hippocampus and underwent RNA‐Seq and RRBS.

**File S1:** Human vs Mouse Overlap and GO.

**File S2:** Genotype vs Diet Reverse Sites.

**File S3:** Local CpG Gene Correlation.

**File S4:** Distal CpG Correlation.

**File S5:** DMRs from ECpGs.

**File S6:** Homer KnownGenes.

**File S7:** Genotype PACs.

**File S8:** Amyloid by Diet PACs.

## Data Availability

The data that support the findings of this study are available in the S2 of this article. Browser tracks are available at the UCSC genome browser at https://genome.ucsc.edu/s/krunican/mm10_AppNLGF_DMCs, containing differentially methylated cytosines (track default setting “dense”, see track description) and https://genome.ucsc.edu/s/krunican/mm10%20AppNLGF%20ECpGs containing expression‐linked CpGs (track default setting “pack”, see track description).
