# Supplementary material for: Brain DNA Methylation Atlas of AppNL‐G‐F Alzheimer's Disease Model Mice Across Age and Region Reveals Choline‐Induced Resilience
Source: Aging Cell. 2025 Oct 13;24(11):e70241. doi: 10.1111/acel.70241 (PMC12611327; doi:10.1111/acel.70241)
Supplement: Supplementary file 1 — Figure S1: Annotations of DMCs changed in AppNL‐G‐F mice versus WT. DMCs from each age point and all ages combined were pooled for downstream annotation. (A) Genomic context annotations for DMCs in the cortex and hippocampus shows primarily promoter enrichment. (B) Distance to nearest transcription start site (TSS) for DMCs in the cortex and hippocampus. (C) Distribution of DMCs over time in the cortex colored by direction of change. (D) Distribution of DMCs over time in the hippocampus colored by direction of change. Depth in Circos plot represents density of DMCs in a region. Figure S2: MeDeCom/DecompPipeline cell type deconvolution can identify major cell populations. Heatmaps showing hierarchical clustering of samples based on cell type proportion in (A) cortex and (B) hippocampus reveals separation between genotypes (n = 96). Comparison between genotypes in the (C) cortex and (D) hippocampus shows cell proportions changed in App NL‐G‐F mice. (E) LMC2 in the cortex correlates with the RNA expression of neuronal gene Glra2 (Spearman‘s ρ = 0.822, p < 0.0001). (F) LMC3 in the cortex correlates with the RNA expression of microglial gene Cd74 (Spearman‘s ρ = 0.657, p < 0.0001). (G) LMC2 in the hippocampus correlates with the RNA expression of neuronal immediate early gene Egr3 (Spearman‘s ρ = 0.67, p < 0.0001). (H) LMC6 in the hippocampus correlates with the expression of glial protein C3 (Spearman‘s ρ = 0.569, p < 0.0001). Data shown as mean ± SEM. Figure S3: Coverage is consistent between brain region batches in AppNL‐G‐F mice versus WT. (A–E) Coverage between all CpGs with a 10‐fold read depth in at least 50% of samples at each age between brain regions in App NL‐G‐F mice versus WT. Figure S4: Overlap between differentially methylated cytosines and total coverage by age in AppNL‐G‐F mice versus WT Overlap between DMCs in the (A) cortex and (B) hippocampus. Comparison of coverage at 10‐fold read depth in at least 50% of samples for DMCs changed in App NL‐G‐F mice v [file ACEL-24-e70241-s007.pdf]

Fig S1

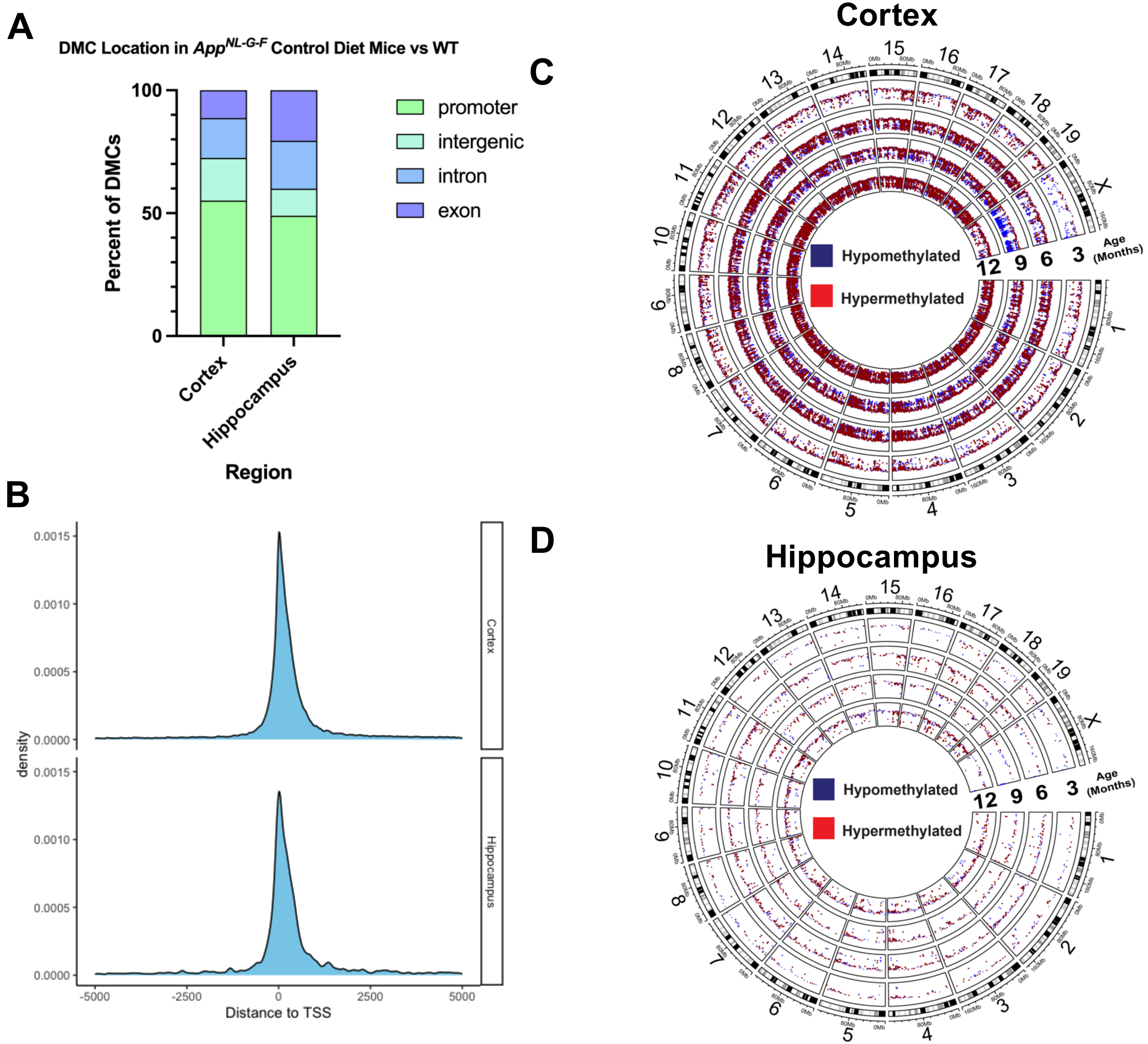
A
DMC Location in App^NL-G-F Control Diet Mice vs WT
Percent of DMCs
100
50
0
promoter
intergenic
intron
exon
Cortex
Hippocampus
Region
B
density
0.0015
0.0010
0.0005
0.0000
Cortex
Hippocampus
-5000
-2500
0
2500
5000
Distance to TSS
C
Cortex
Hypomethylated
Hypermethylated
12 9 6 3
Age (Months)
D
Hippocampus
Hypomethylated
Hypermethylated
12 9 6 3
Age (Months)

Fig S2

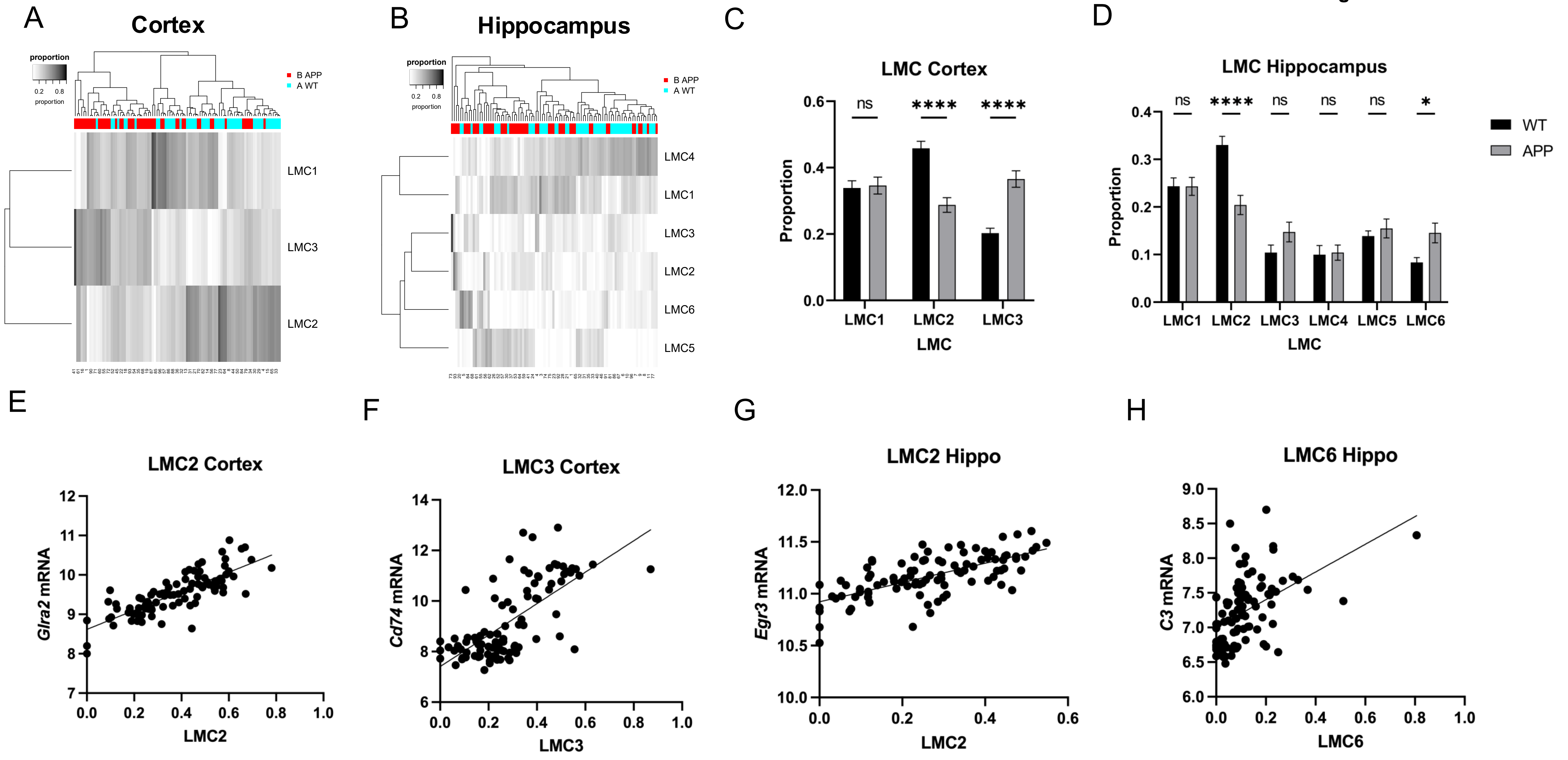

A
Cortex
proportion
0.2 0.8
B APP
A WT
LMC1
LMC3
LMC2
B
Hippocampus
proportion
0.2 0.8
B APP
A WT
LMC4
LMC1
LMC3
LMC2
LMC6
LMC5
C
LMC Cortex
ns
****
****
Proportion
0.0
0.2
0.4
0.6
LMC1
LMC2
LMC3
LMC
D
LMC Hippocampus
ns
****
ns
ns
ns
*
Proportion
0.0
0.1
0.2
0.3
0.4
LMC1
LMC2
LMC3
LMC4
LMC5
LMC6
LMC
WT
APP
E
LMC2 Cortex
Glra2 mRNA
LMC2
F
LMC3 Cortex
Cd74 mRNA
LMC3
G
LMC2 Hippo
Egr3 mRNA
LMC2
H
LMC6 Hippo
C3 mRNA
LMC6

Fig S3

Coverage in $App^{NL\text{-}G\text{-}F}$ vs WT

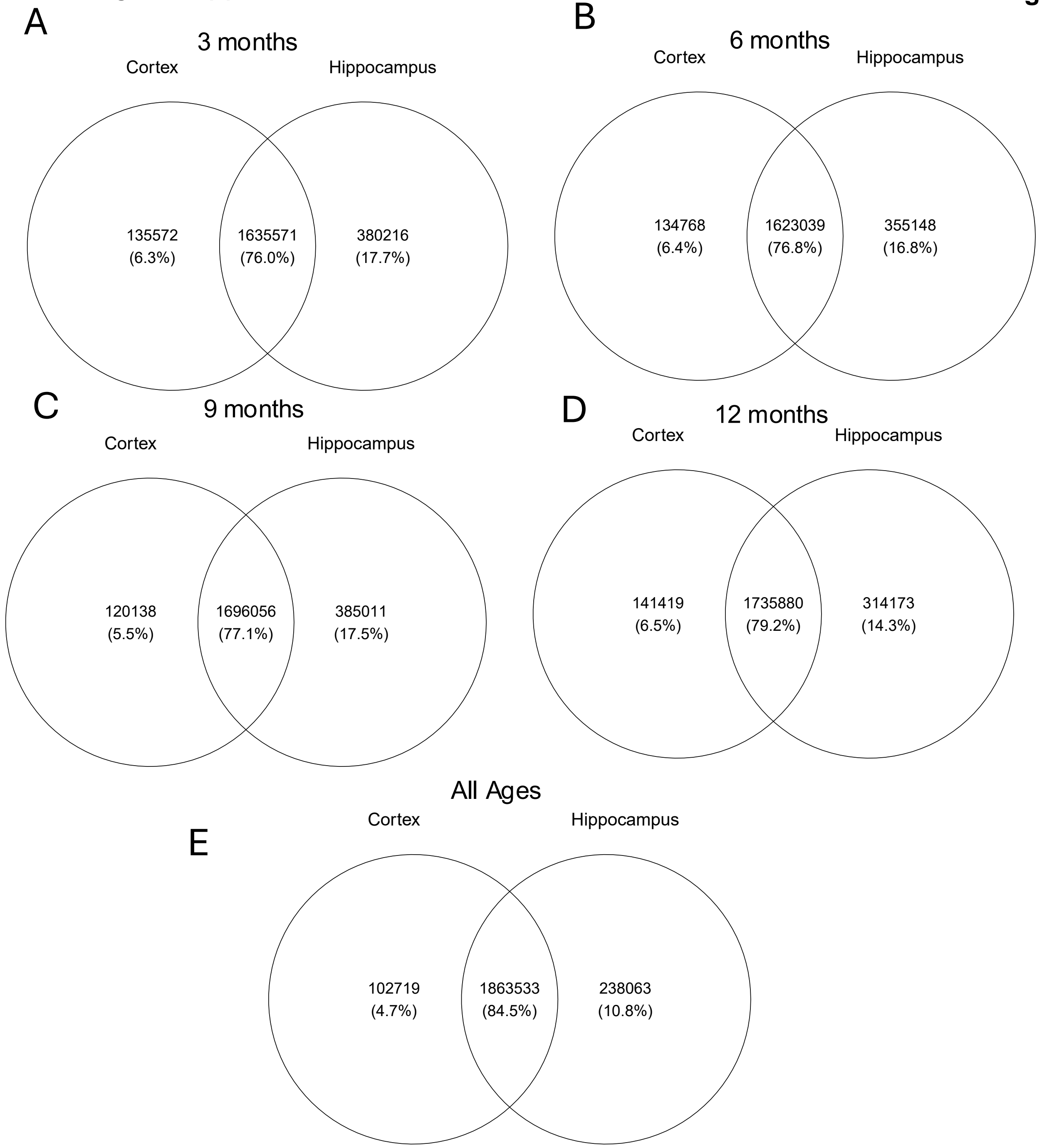

Cortex ***App$^{NL\text{-}G\text{-}F}$*** **vs WT Control Diet** **Hippocampus** **Fig S4**

Differentially Methylated Cytosines

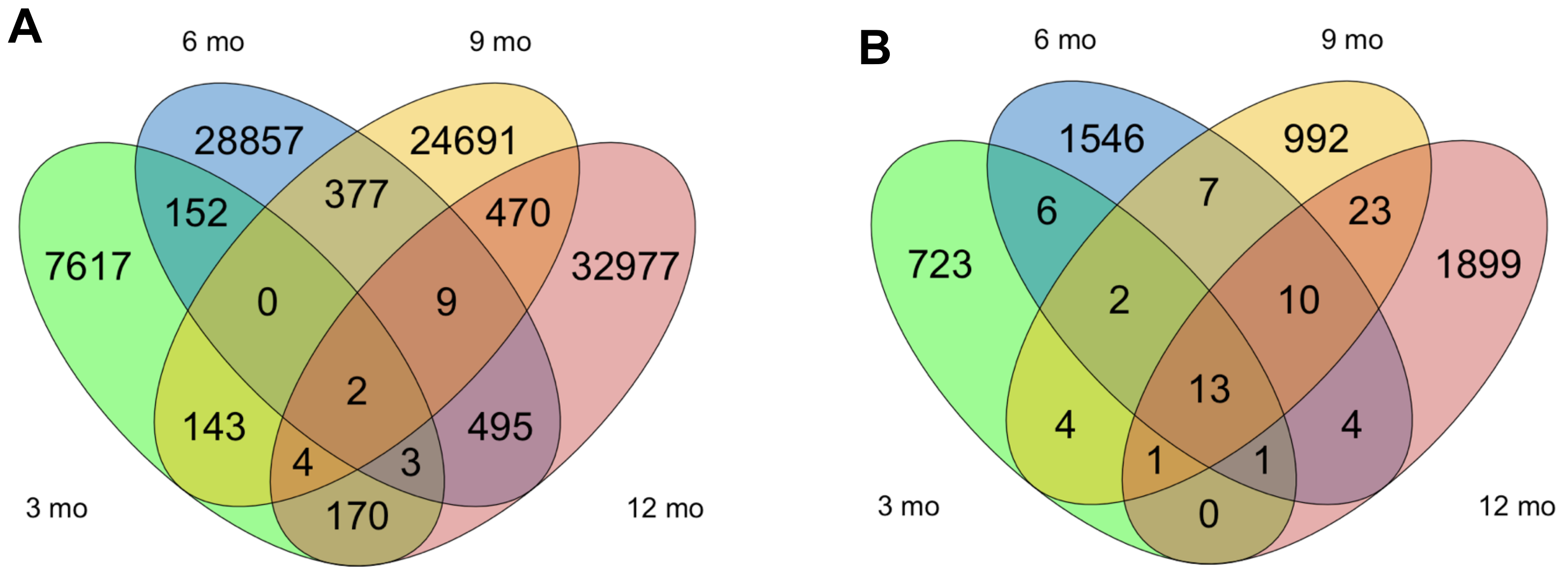

Coverage

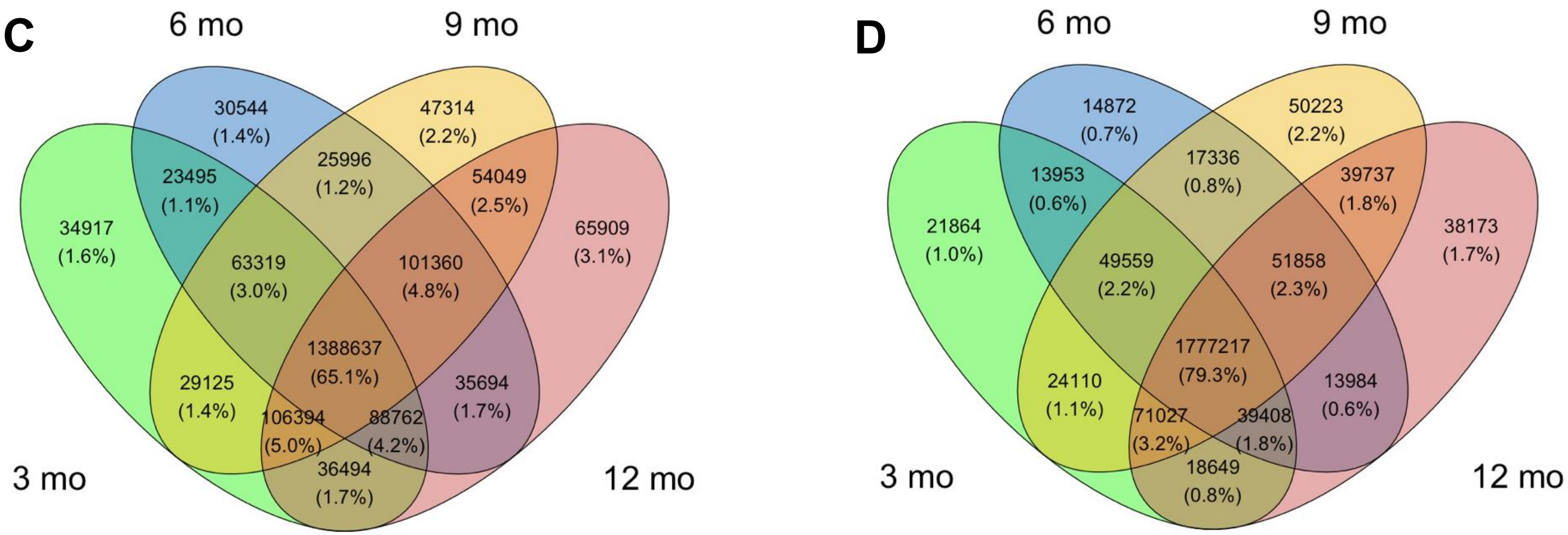

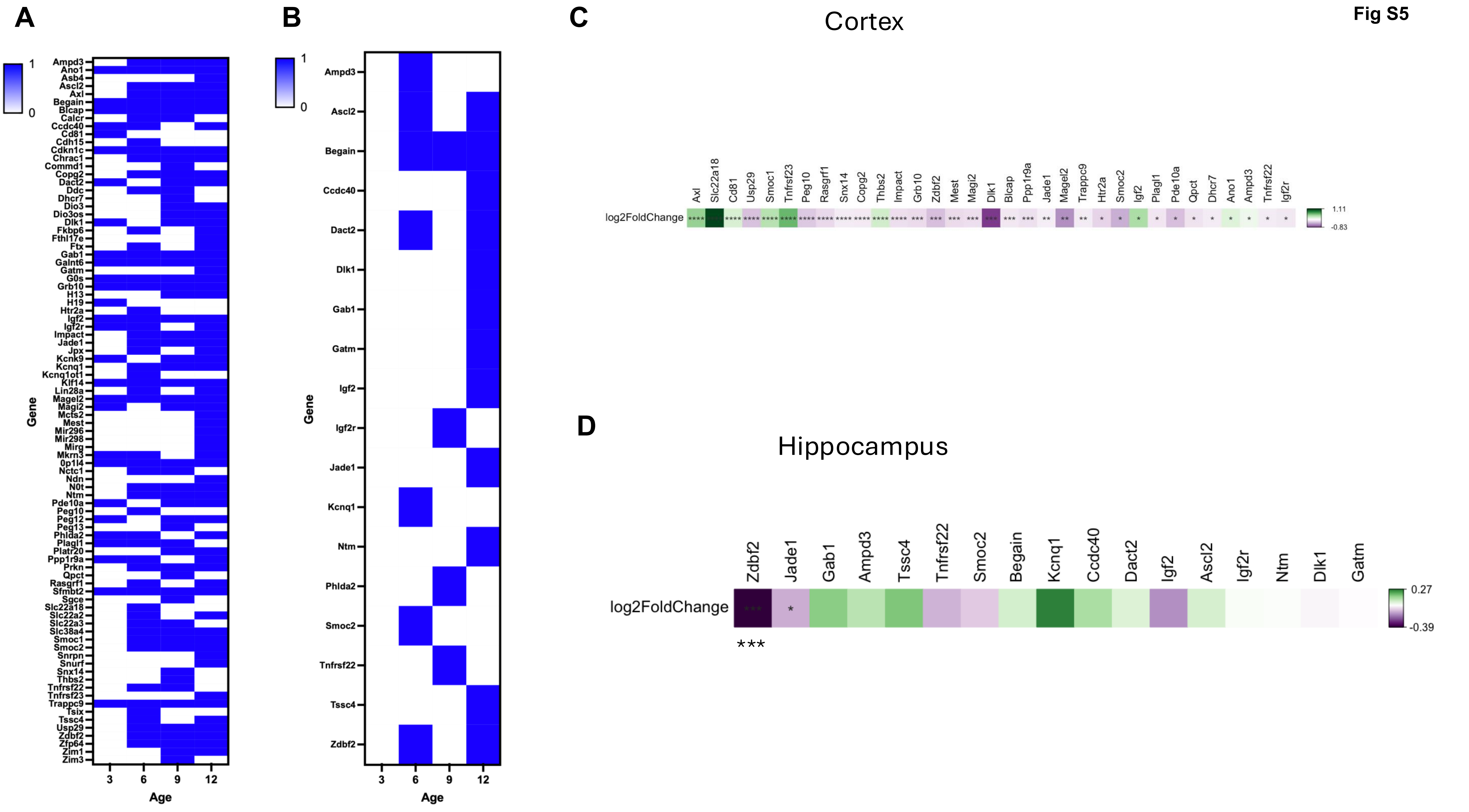

Fig S5
A
B
C
D
Cortex
Hippocampus
Gene
Age
log2FoldChange
Ampd3
Ascl2
Begain
Ccdc40
Dact2
Dlk1
Gab1
Gatm
Igf2
Igf2r
Jade1
Kcnq1
Ntm
Phlda2
Smoc2
Tnfrsf22
Tssc4
Zdbf2

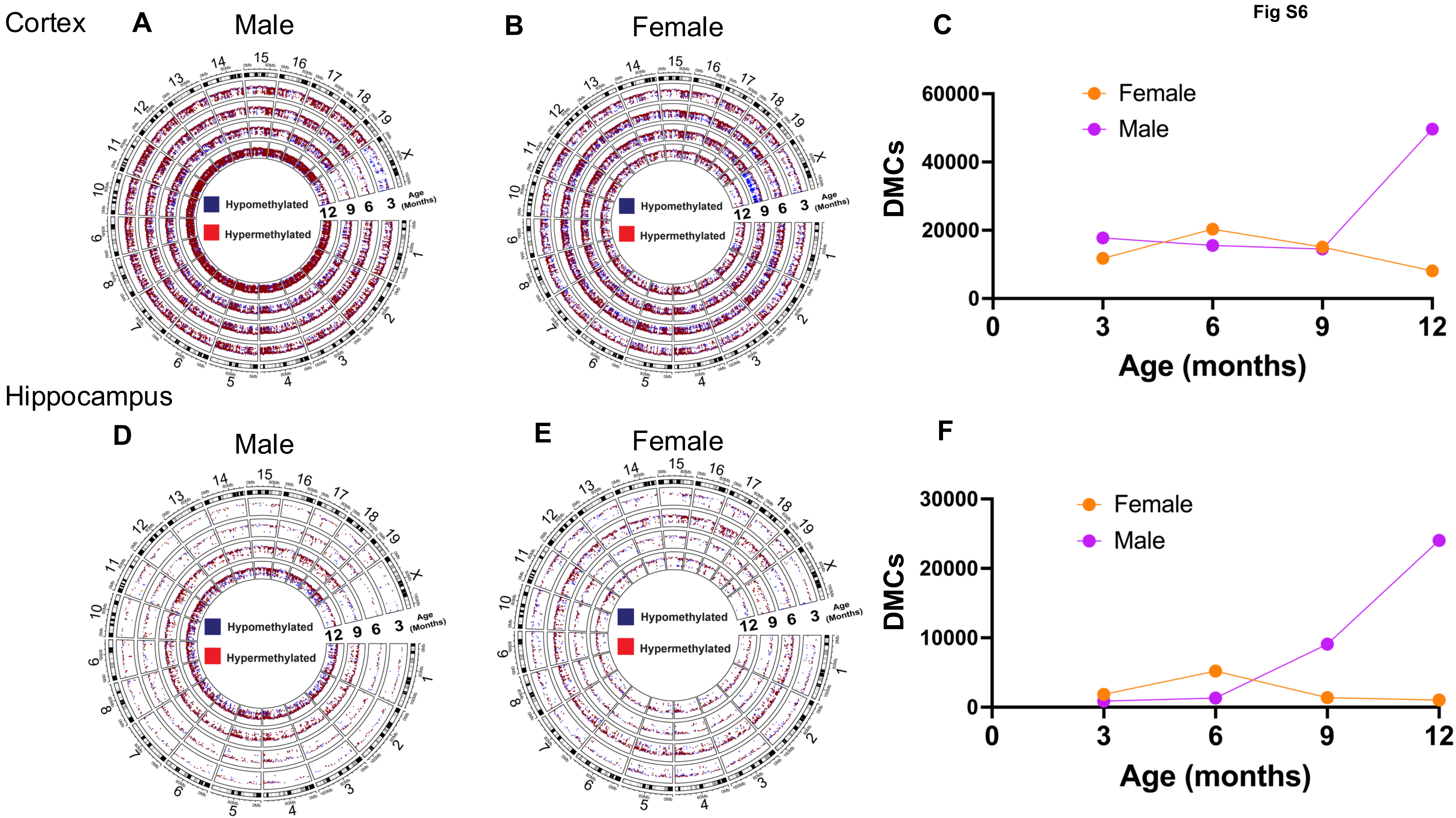

Fig S6
Cortex
A
Male
B
Female
C
Hypomethylated
Hypermethylated
Age (Months)
Female
Male
DMCs
Age (months)
Hippocampus
D
Male
E
Female
F

Fig S7

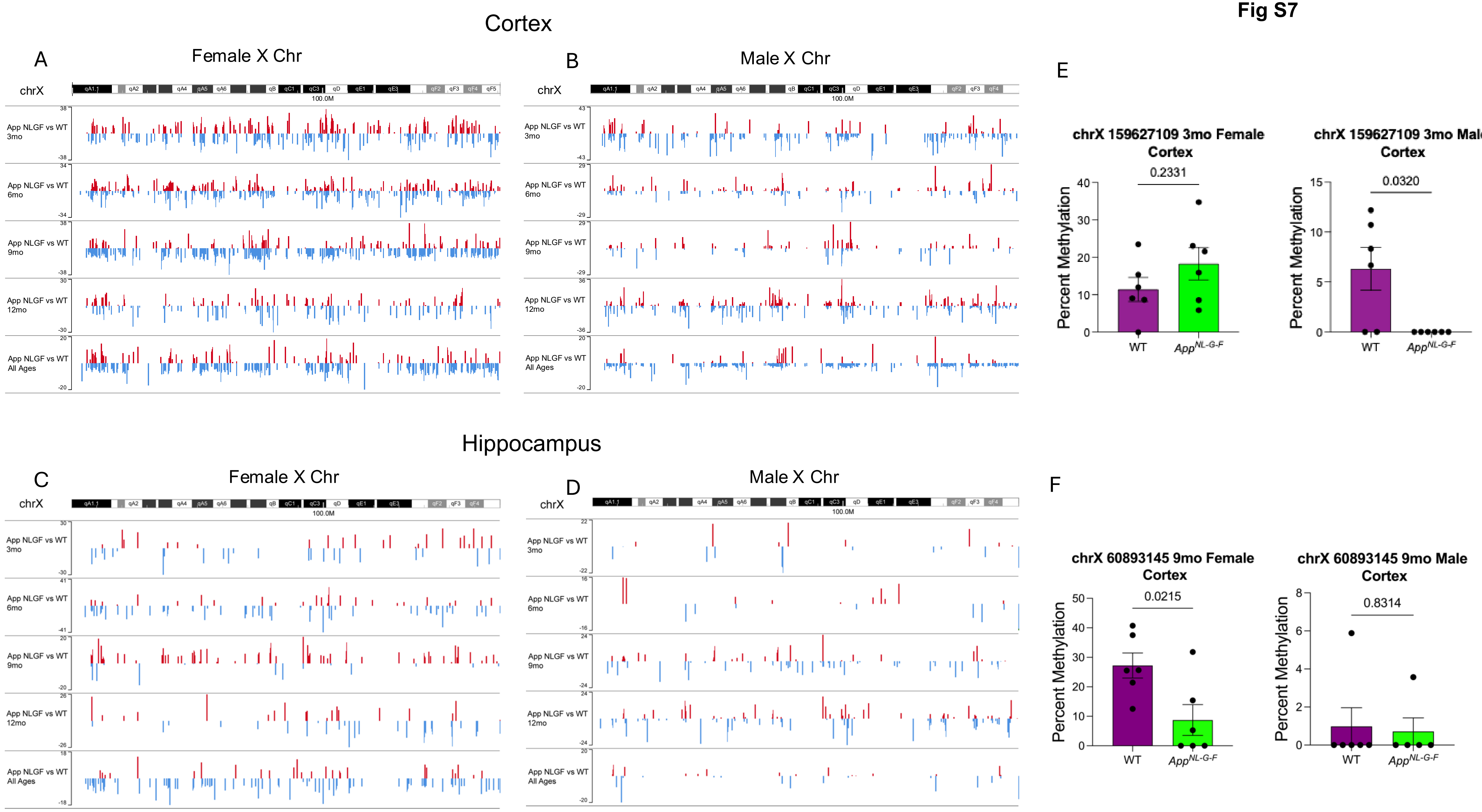

Cortex
A
Female X Chr
B
Male X Chr
chrX
100.0M
App NLGF vs WT 3mo
App NLGF vs WT 6mo
App NLGF vs WT 9mo
App NLGF vs WT 12mo
App NLGF vs WT All Ages
E
chrX 159627109 3mo Female Cortex
0.2331
chrX 159627109 3mo Male Cortex
0.0320
Percent Methylation
WT
App NL-G-F
Hippocampus
C
Female X Chr
D
Male X Chr
F
chrX 60893145 9mo Female Cortex
0.0215
chrX 60893145 9mo Male Cortex
0.8314

Fig S8

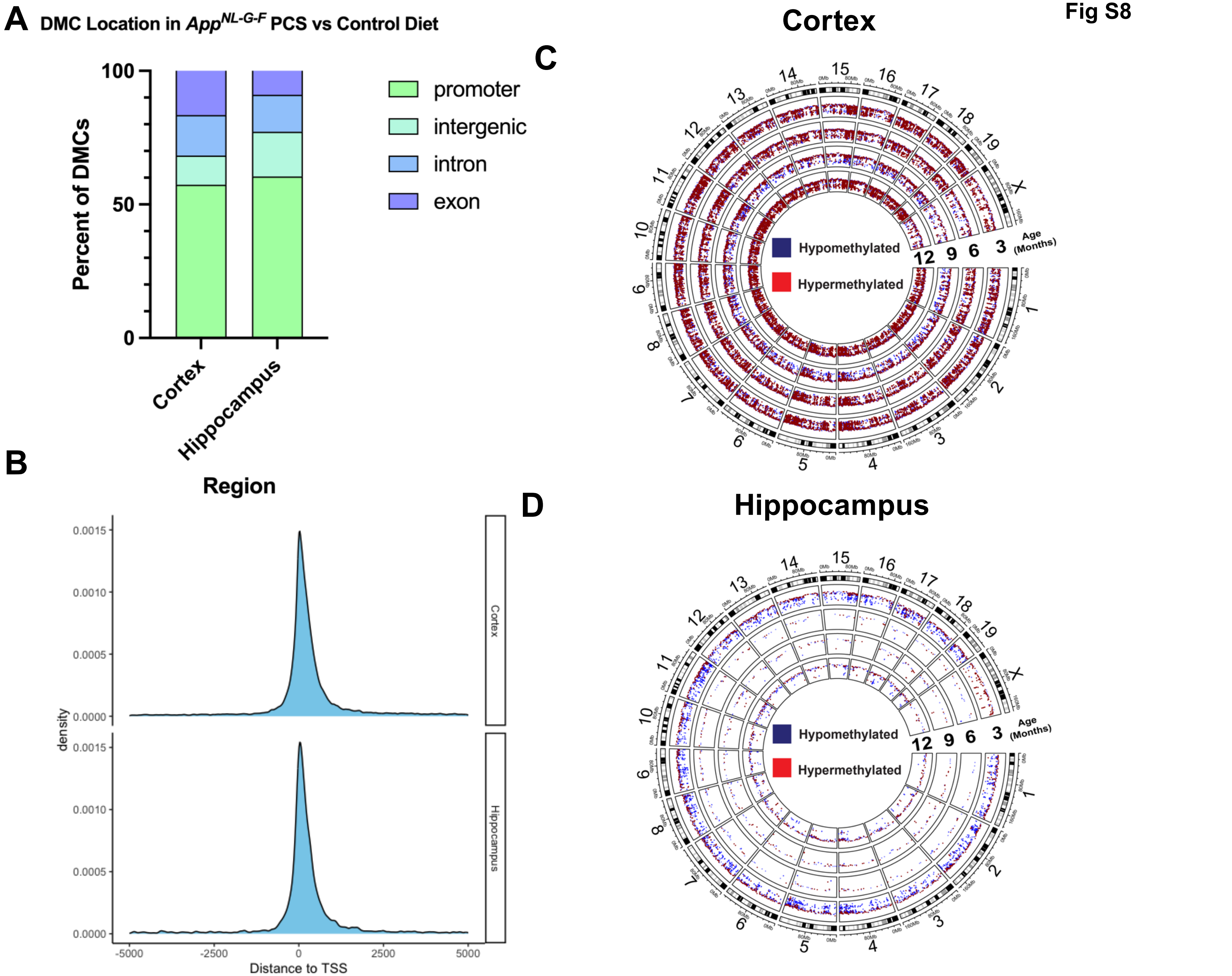
A DMC Location in AppNL-G-F PCS vs Control Diet
Percent of DMCs
100
50
0
Cortex
Hippocampus
promoter
intergenic
intron
exon
B
Region
density
0.0015
0.0010
0.0005
0.0000
Cortex
Hippocampus
-5000
-2500
0
2500
5000
Distance to TSS
C
Cortex
Hypomethylated
Hypermethylated
12
9
6
3
Age (Months)
D
Hippocampus
Hypomethylated
Hypermethylated
12
9
6
3
Age (Months)

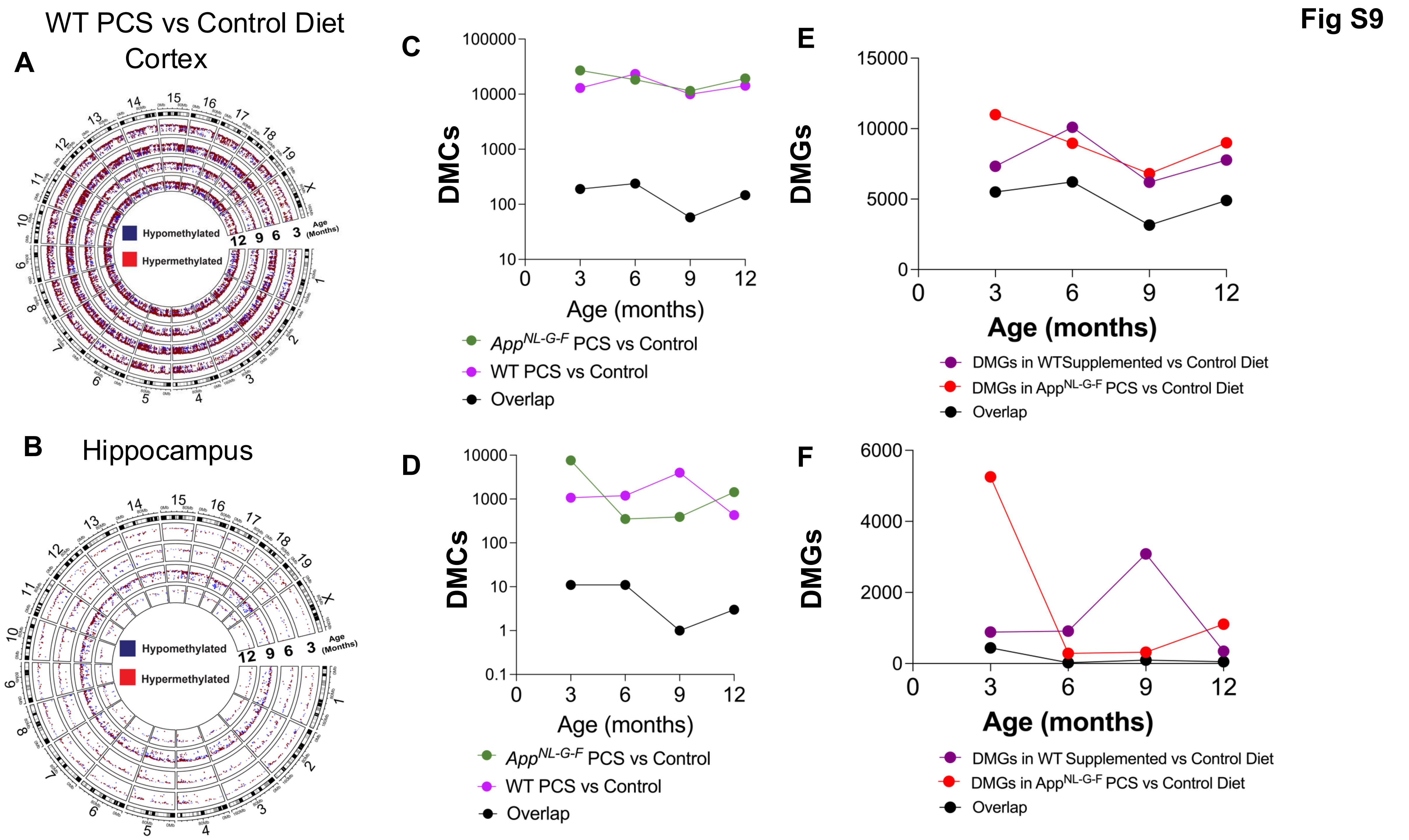
Fig S9
WT PCS vs Control Diet
A
Cortex
Hypomethylated
Hypermethylated
Age (Months)
12 9 6 3
B
Hippocampus
C
DMCs
Age (months)
App^NL-G-F PCS vs Control
WT PCS vs Control
Overlap
D
E
DMGs
DMGs in WTSupplemented vs Control Diet
DMGs in App^NL-G-F PCS vs Control Diet
F
DMGs in WT Supplemented vs Control Diet

Fig S10

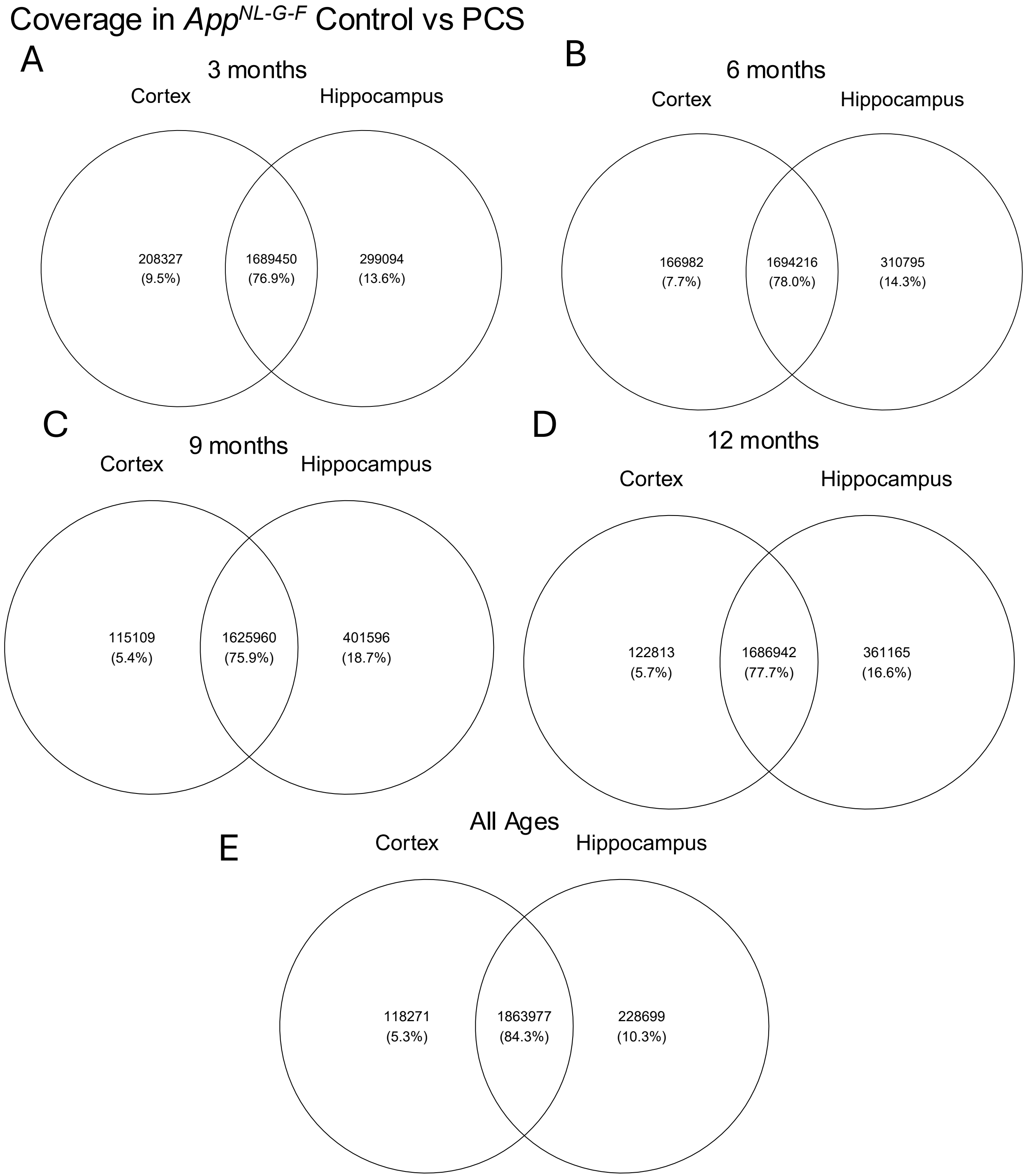
Coverage in App^{NL-G-F} Control vs PCS
A
3 months
Cortex
Hippocampus
208327
(9.5%)
1689450
(76.9%)
299094
(13.6%)
B
6 months
Cortex
Hippocampus
166982
(7.7%)
1694216
(78.0%)
310795
(14.3%)
C
9 months
Cortex
Hippocampus
115109
(5.4%)
1625960
(75.9%)
401596
(18.7%)
D
12 months
Cortex
Hippocampus
122813
(5.7%)
1686942
(77.7%)
361165
(16.6%)
E
All Ages
Cortex
Hippocampus
118271
(5.3%)
1863977
(84.3%)
228699
(10.3%)

Cortex ***App$^{NL\text{-}G\text{-}F}$* vs WT Control Diet** **Hippocampus** **Fig S11**

Differentially Methylated Cytosines

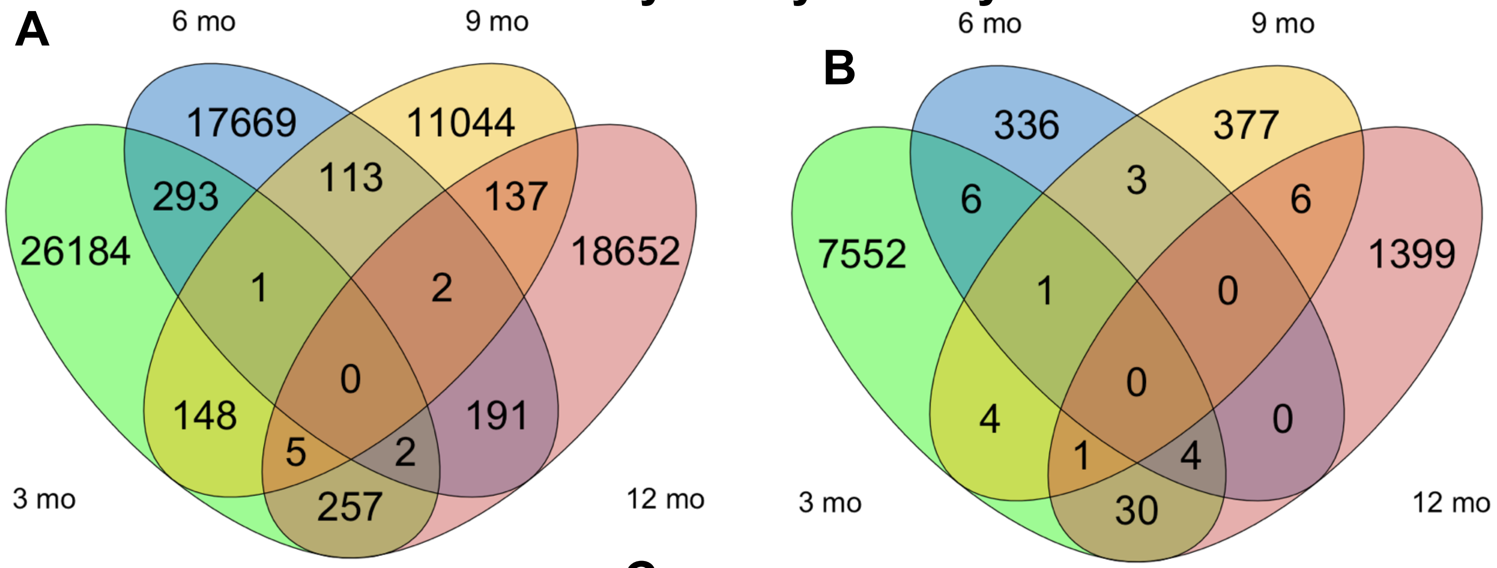

Coverage

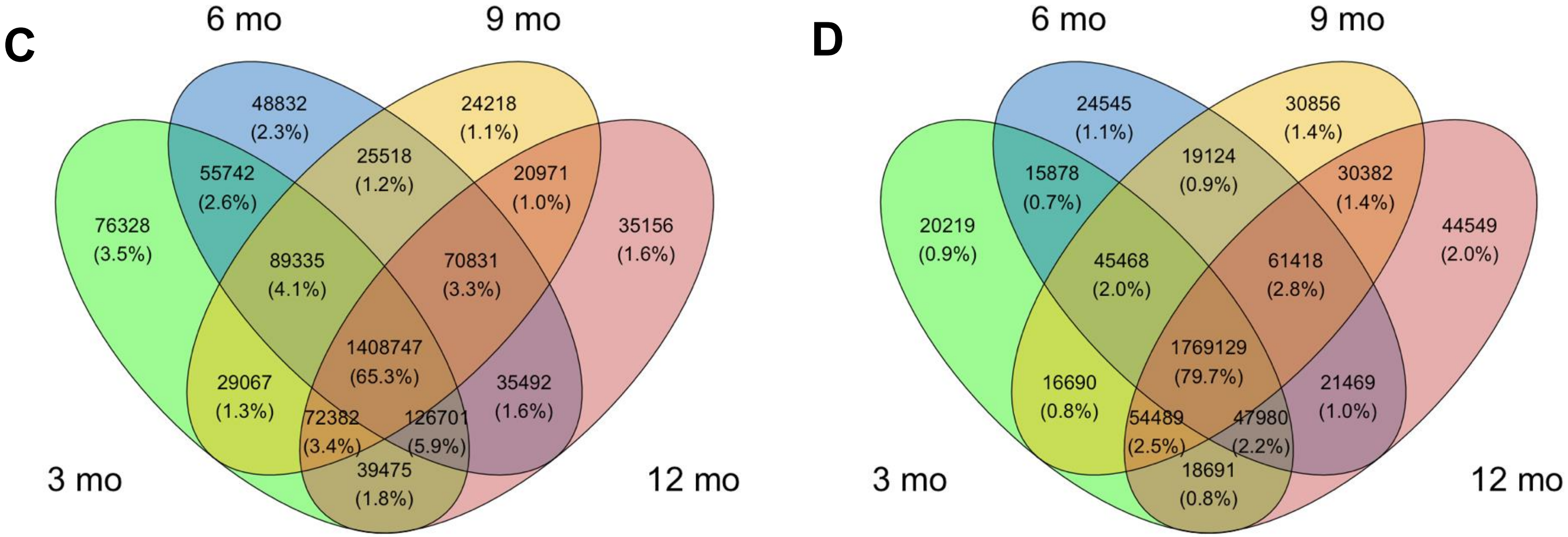

Fig S12

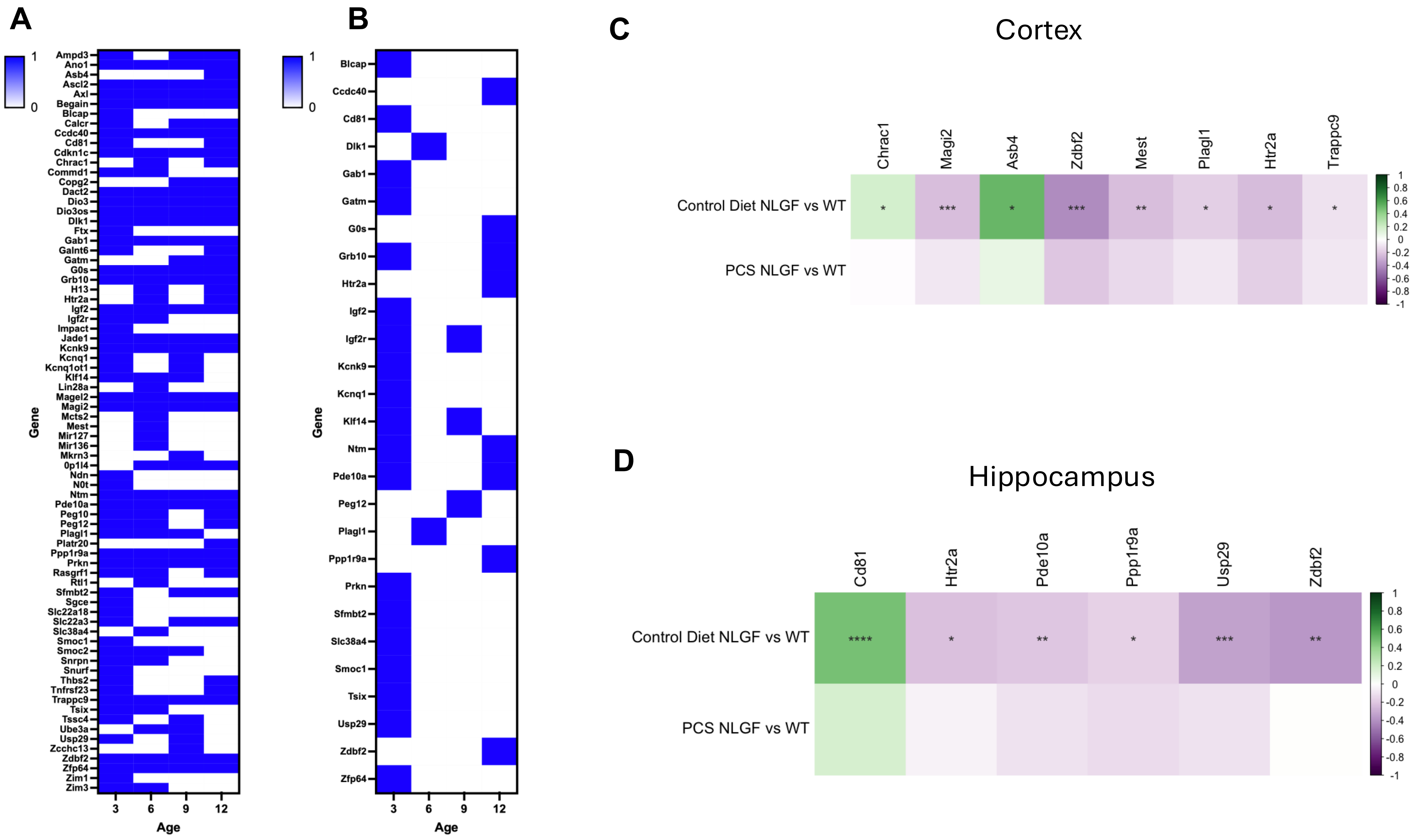
A
B
C
Cortex
D
Hippocampus
Gene
Age
3
6
9
12
Control Diet NLGF vs WT
PCS NLGF vs WT
Chrac1
Magi2
Asb4
Zdbf2
Mest
Plagl1
Htr2a
Trappc9
Cd81
Pde10a
Ppp1r9a
Usp29

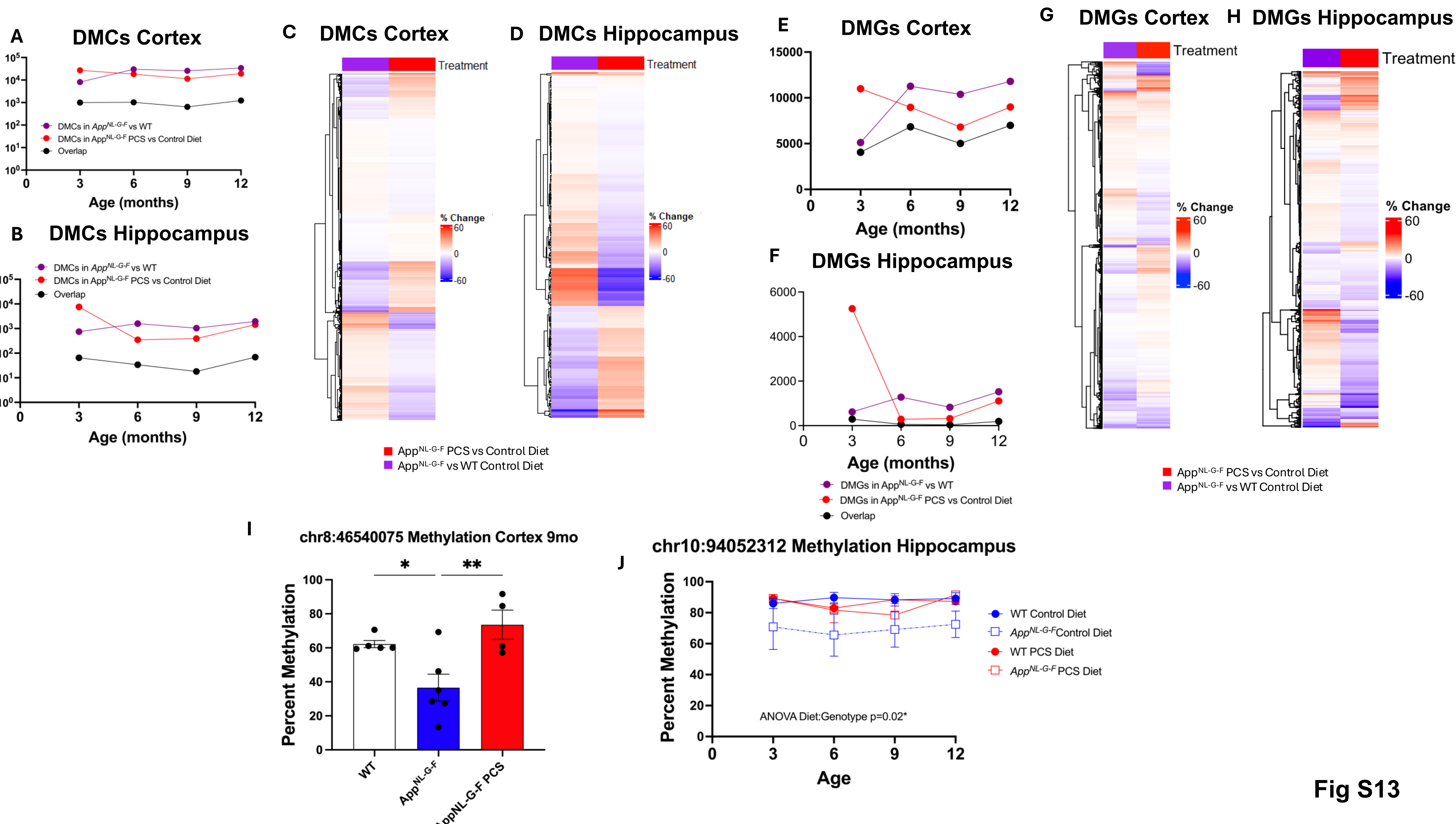

A
DMCs Cortex
DMCs in AppNL-G-F vs WT
DMCs in AppNL-G-F PCS vs Control Diet
Overlap
Age (months)
B
DMCs Hippocampus
DMCs in AppNL-G-F vs WT
DMCs in AppNL-G-F PCS vs Control Diet
Overlap
Age (months)
C
DMCs Cortex
Treatment
% Change
D
DMCs Hippocampus
Treatment
% Change
AppNL-G-F PCS vs Control Diet
AppNL-G-F vs WT Control Diet
E
DMGs Cortex
Age (months)
F
DMGs Hippocampus
Age (months)
DMGs in AppNL-G-F vs WT
DMGs in AppNL-G-F PCS vs Control Diet
Overlap
G
DMGs Cortex
Treatment
% Change
H
DMGs Hippocampus
Treatment
% Change
AppNL-G-F PCS vs Control Diet
AppNL-G-F vs WT Control Diet
I
chr8:46540075 Methylation Cortex 9mo
Percent Methylation
WT
AppNL-G-F
AppNL-G-F PCS
J
chr10:94052312 Methylation Hippocampus
Percent Methylation
ANOVA Diet:Genotype p=0.02*
Age
WT Control Diet
AppNL-G-F Control Diet
WT PCS Diet
AppNL-G-F PCS Diet

Fig S13

Fig S14

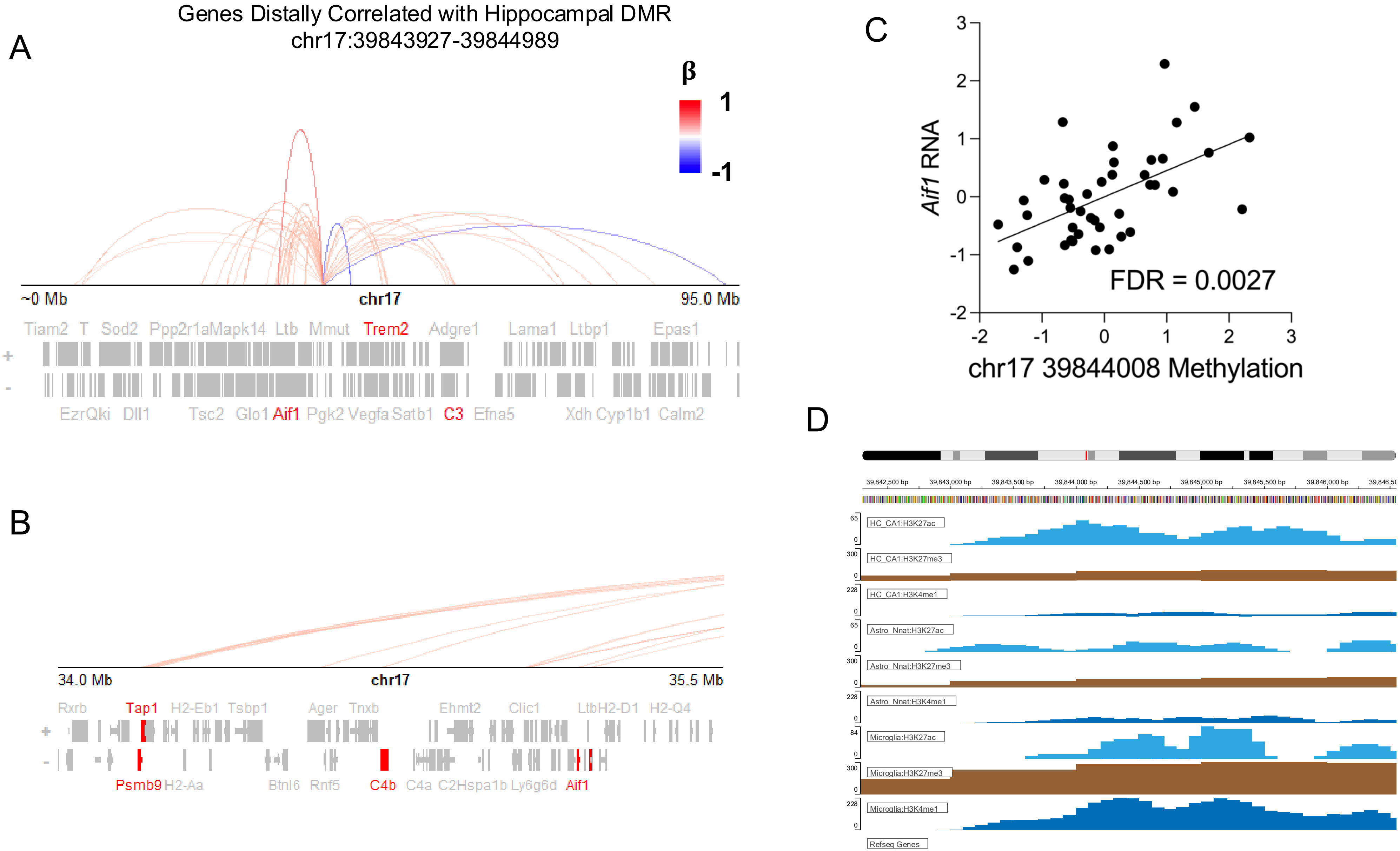
A
Genes Distally Correlated with Hippocampal DMR
chr17:39843927-39844989
β
1
-1
~0 Mb
chr17
95.0 Mb
Tiam2 T Sod2 Ppp2r1aMapk14 Ltb Mmut Trem2 Adgre1 Lama1 Ltbp1 Epas1
EzrQki Dll1 Tsc2 Glo1 Aif1 Pgk2 Vegfa Satb1 C3 Efna5 Xdh Cyp1b1 Calm2
B
34.0 Mb
chr17
35.5 Mb
Rxrb Tap1 H2-Eb1 Tsbp1 Ager Tnxb Ehmt2 Clic1 LtbH2-D1 H2-Q4
Psmb9 H2-Aa Btnl6 Rnf5 C4b C4a C2Hspa1b Ly6g6d Aif1
C
Aif1 RNA
chr17 39844008 Methylation
FDR = 0.0027
D
HC_CA1:H3K27ac
HC_CA1:H3K27me3
HC_CA1:H3K4me1
Astro_Nnat:H3K27ac
Astro_Nnat:H3K27me3
Astro_Nnat:H3K4me1
Microglia:H3K27ac
Microglia:H3K27me3
Microglia:H3K4me1
Refseq Genes

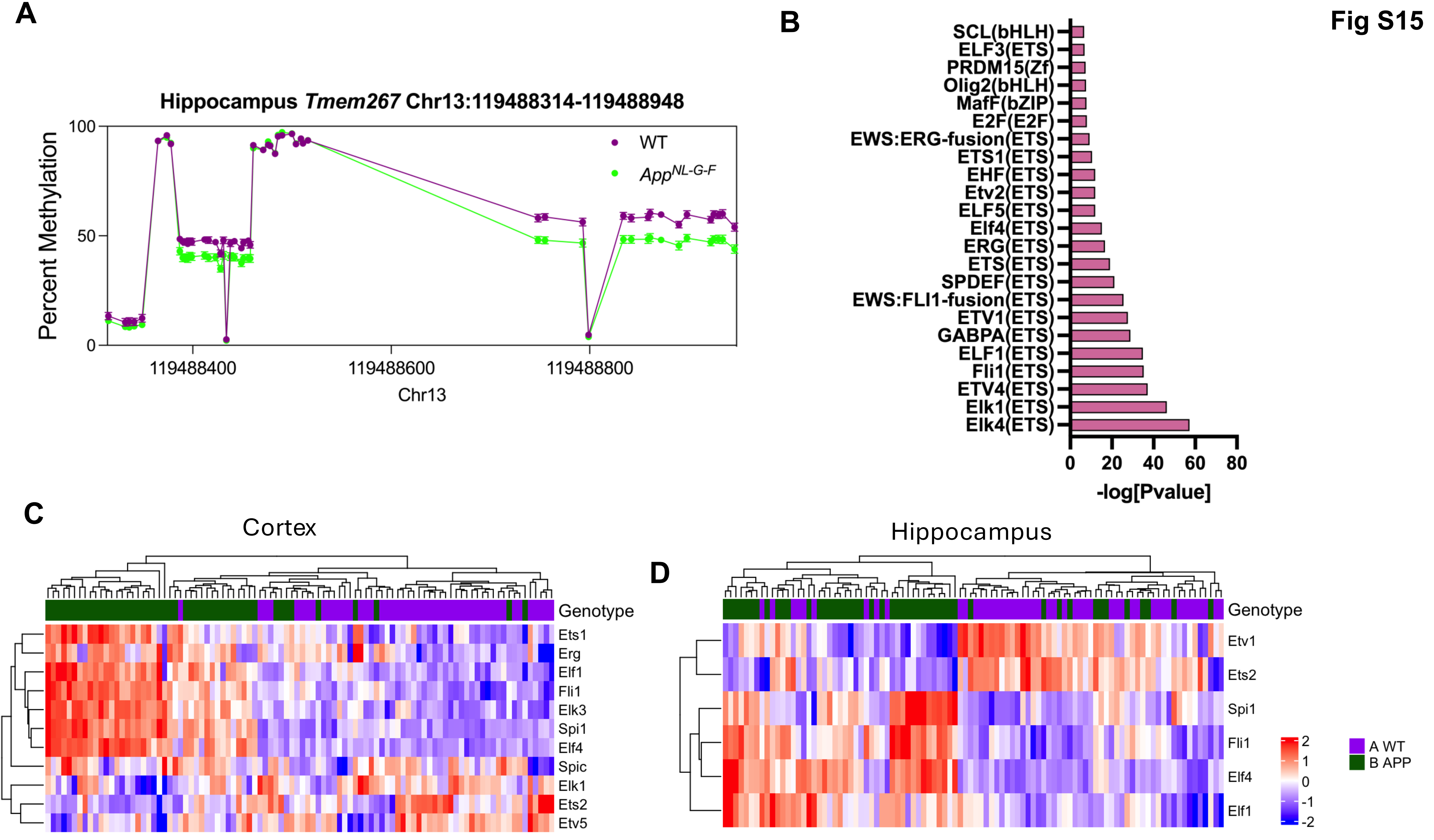
Fig S15
A
Hippocampus Tmem267 Chr13:119488314-119488948
Percent Methylation
100
50
0
WT
App^NL-G-F
119488400
119488600
119488800
Chr13
B
SCL(bHLH)
ELF3(ETS)
PRDM15(Zf)
Olig2(bHLH)
MafF(bZIP)
E2F(E2F)
EWS:ERG-fusion(ETS)
ETS1(ETS)
EHF(ETS)
Etv2(ETS)
ELF5(ETS)
Elf4(ETS)
ERG(ETS)
ETS(ETS)
SPDEF(ETS)
EWS:FLI1-fusion(ETS)
ETV1(ETS)
GABPA(ETS)
ELF1(ETS)
Fli1(ETS)
ETV4(ETS)
Elk1(ETS)
Elk4(ETS)
0 20 40 60 80
-log[Pvalue]
C
Cortex
Genotype
Ets1
Erg
Elf1
Fli1
Elk3
Spi1
Elf4
Spic
Elk1
Ets2
Etv5
D
Hippocampus
Genotype
Etv1
Ets2
Spi1
Fli1
Elf4
Elf1
2
1
0
-1
-2
A WT
B APP

Fig S16

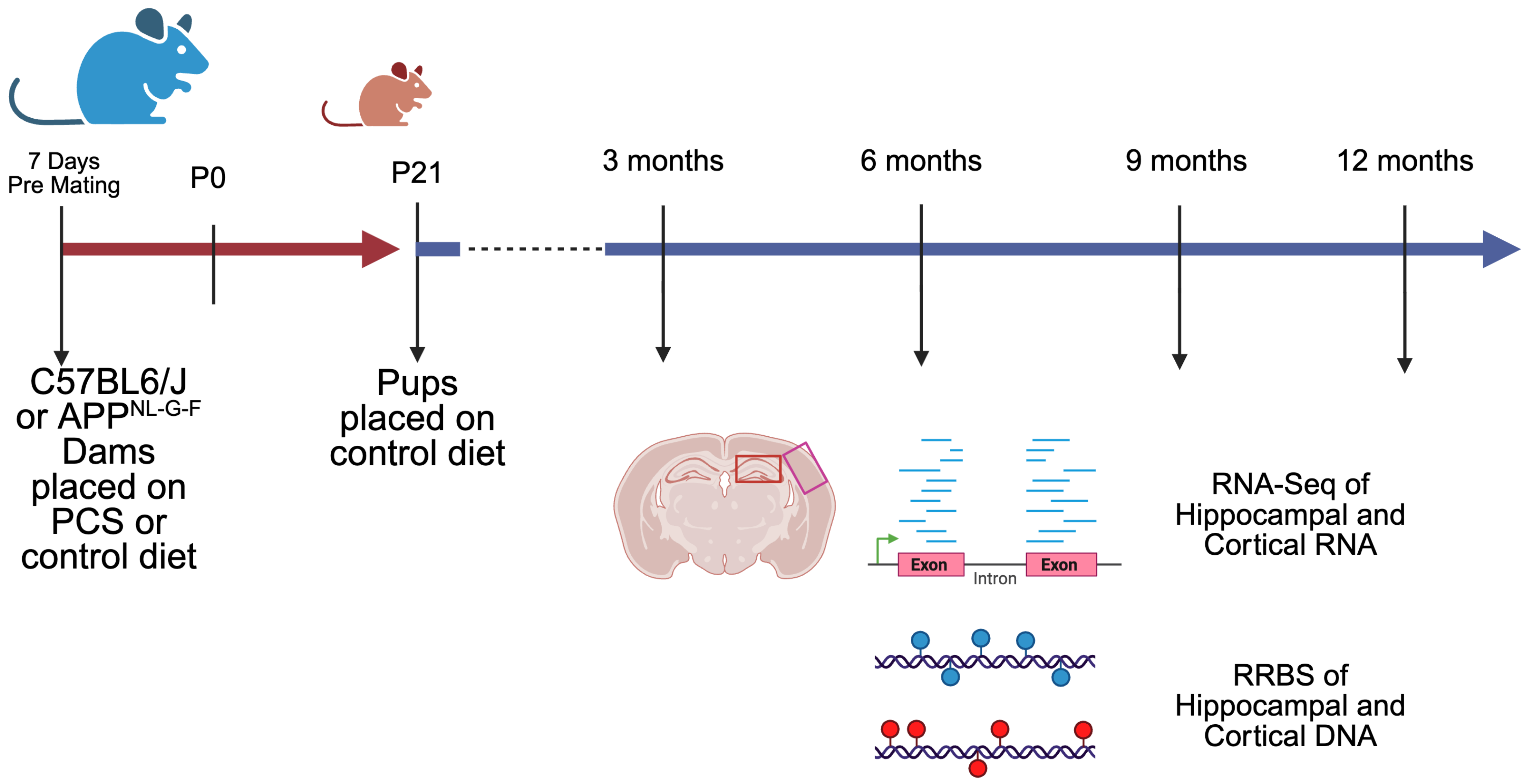
7 Days
Pre Mating
P0
P21
3 months
6 months
9 months
12 months
C57BL6/J
or APP^NL-G-F
Dams
placed on
PCS or
control diet
Pups
placed on
control diet
Exon
Intron
Exon
RNA-Seq of
Hippocampal and
Cortical RNA
RRBS of
Hippocampal and
Cortical DNA

Supplemental Figures and Files

Figure S1: Annotations of DMCs changed in *App*$^{NL\text{-}G\text{-}F}$ mice versus WT
DMCs from each age point and all ages combined were pooled for downstream annotation. **A)** Genomic context annotations for DMCs in the cortex and hippocampus shows primarily promoter enrichment. **B)** Distance to nearest transcription start site (TSS) for DMCs in the cortex and hippocampus. **C)** Distribution of DMCs over time in the cortex colored by direction of change. **D)** Distribution of DMCs over time in the hippocampus colored by direction of change. Depth in Circos plot represents density of DMCs in a region.

Figure S2: MeDeCom/DecompPipeline cell type deconvolution can identify major cell populations Heatmaps showing hierarchical clustering of samples based on cell type proportion in **A)** cortex and **B)** hippocampus reveals separation between genotypes (n = 96). Comparison between genotypes in the **C)** cortex and **D)** hippocampus shows cell proportions changed in *App*$^{NL\text{-}G\text{-}F}$ mice. **E)** LMC2 in the cortex correlates with the RNA expression of neuronal gene *Glra2* (Spearman's $\rho = 0.822$, $p < 0.0001$). **F)** LMC3 in the cortex correlates with the RNA expression of microglial gene *Cd74 (*Spearman's $\rho = 0.657$, $p < 0.0001$). **G)** LMC2 in the hippocampus correlates with the RNA expression of neuronal immediate early gene *Egr3 (*Spearman's $\rho = 0.67$, $p < 0.0001$). **H)** LMC6 in the hippocampus correlates with the expression of glial protein *C3 (*Spearman's $\rho = 0.569$, $p < 0.0001$). Data shown as mean ± SEM.

Figure S3: Coverage is consistent between brain region batches in *App*$^{NL\text{-}G\text{-}F}$ mice versus WT
A-E) Coverage between all CpGs with a 10-fold read depth in at least 50% of samples at each age between brain regions in *App*$^{NL\text{-}G\text{-}F}$ mice versus WT.

Figure S4: Overlap between differentially methylated cytosines and total coverage by age in *App*$^{NL\text{-}G\text{-}F}$ mice versus WT
Overlap between DMCs in the **A)** cortex and **B)** hippocampus. Comparison of coverage at 10-fold read depth in at least 50% of samples for DMCs changed in *App*$^{NL\text{-}G\text{-}F}$ mice versus WT in the **C)** cortex and **D)** hippocampus.

Figure S5: *App*$^{NL\text{-}G\text{-}F}$ mice show differential methylation of imprinted genes compared to WT
DMCs at each age were mapped to their nearest gene as described in the methods. Differential methylation of imprinted genes shown in the **A)** cortex and **B)** hippocampus. Blue represents differentially methylated in *App*$^{NL\text{-}G\text{-}F}$ mice compared to WT. **C-D)** Heatmap showcasing RNA expression log2 fold changes denoted with the color scale and adjusted p values for imprinted genes in the cortex and hippocampus. *$p < 0.05$, **$p < 0.01$, ***$p < 0.001$, ****$p < 0.0001$.

Figure S6: Whole genome DMCs in *App*$^{NL\text{-}G\text{-}F}$ mice versus WT stratified by sex
Circos plots showing genome-wide distribution of DMCs in **A)** male and **B)** female cortex. **C)** Quantification of DMCs over time in male and female cortex. Circos plots showing genome-wide distribution of DMCs in **D)** male and **E)** female hippocampus. **F)** Quantification of DMCs over time in male and female hippocampus.

Figure S7: *App$^{NL\text{-}G\text{-}F}$* mice show sex dependent X chromosome methylation compared to wild type

Reduced representation bisulfite sequencing was performed on the hippocampus and cortex of male or female *App$^{NL\text{-}G\text{-}F}$* or C57BL/6J mice, controlling for age and diet (n=24 per sex per genotype, n = 48 total). CpGs with < 0.05 q-value were considered differentially methylated. X chromosome diagrams showing DMC percent change in *App$^{NL\text{-}G\text{-}F}$* vs WT mice in the cortex by age in **A)** females and **B)** males. **C)** Diagrams showing DMC percent change in *App$^{NL\text{-}G\text{-}F}$* vs WT mice in the hippocampus by age in **C)** females and **D)** males. **E)** ChrX:159627109 (located within the *Sh3kbp1* gene) is hypomethylated in male *App$^{NL\text{-}G\text{-}F}$* mice at 3 months (Welch-corrected t = 2.947, p = 0.032). ChrX:159627109 methylation does not change at 3 months in the cortex of female *App$^{NL\text{-}G\text{-}F}$* mice (Welch-corrected t = 1.276, p = 0.2331). Data shown as mean ± SEM. **F)** ChrX:60893145 (located within the *Sox3* gene) is hypomethylated in female *App$^{NL\text{-}G\text{-}F}$* mice at 9 months (Welch-corrected t = 2.743, p = 0.0215). ChrX:60893145 methylation does not change at 9 months in the cortex of male *App$^{NL\text{-}G\text{-}F}$* mice (Welch-corrected t = 0.2194, p = 0.8314).

Figure S8: Annotations of DMCs changed in *App$^{NL\text{-}G\text{-}F}$* PCS versus control diet mice

DMCs from each age point and all ages combined were pooled for downstream annotation. **A)** Genomic context annotations for DMCs in the cortex and hippocampus shows primarily promoter enrichment. **B)** Distance to nearest transcription start site (TSS) for DMCs in the cortex and hippocampus. **C)** Distribution of DMCs over time in the cortex colored by direction of change. **D)** Distribution of DMCs over time in the hippocampus colored by direction of change. Depth in Circos plot represents density of DMCs in a region.

Figure S9: *App$^{NL\text{-}G\text{-}F}$* and WT PCS mice have distinct methylation changes

Circos plots showing genome-wide distribution of DMCs in PCS versus control diet WT mice in **A)** cortex and **B)** hippocampus. Overlap of DMCs between *App$^{NL\text{-}G\text{-}F}$* PCS vs control diet and WT PCS vs control diet shows limited overlap in **C)** cortex and **D)** hippocampus. DMGs were determined by mapping each CpG to its nearest gene as described in the methods. Overlap of DMGs between *App$^{NL\text{-}G\text{-}F}$* PCS vs control diet and WT PCS vs control diet shows high degree of overlap in **E)** cortex that is not present in the **F)** hippocampus.

Figure S10: Coverage is consistent between brain region batches in *App$^{NL\text{-}G\text{-}F}$* PCS vs control diet mice

A-E) Coverage between all CpGs with a 10-fold read depth in at least 50% of samples at each age between brain regions in *App$^{NL\text{-}G\text{-}F}$* PCS vs control diet mice.

Figure S11: Overlap between differentially methylated cytosines and total coverage by age in *App$^{NL\text{-}G\text{-}F}$* PCS vs control diet mice

Overlap between DMCs in the **A)** cortex and **B)** hippocampus. Comparison of coverage at 10-fold read depth in at least 50% of samples for DMCs changed in *App$^{NL\text{-}G\text{-}F}$* PCS versus control diet mice in the **C)** cortex and **D)** hippocampus.

Figure S12: *App$^{NL\text{-}G\text{-}F}$* mice fed a PCS diet show differential DNA methylation of imprinted genes compared to control diet

DMCs at each age were mapped to their nearest gene as described in the methods. Differential methylation of imprinted genes shown in the **A)** cortex and **B)** hippocampus. Blue represents

differentially methylated in PCS *App*$^{NL\text{-}G\text{-}F}$ mice compared to control diet. **C-D)** Heatmap showcasing RNA expression log2 fold changes denoted by the color scale and adjusted p values for imprinted genes in control diet *App*$^{NL\text{-}G\text{-}F}$ vs WT and PCS diet *App*$^{NL\text{-}G\text{-}F}$ vs WT the cortex and hippocampus. *$p < 0.05$, **$p < 0.01$, ***$p < 0.001$, ****$p < 0.0001$.

Figure S13: Perinatal choline supplementation can reverse genotype-induced DNA methylation changes in *App*$^{NL\text{-}G\text{-}F}$ mice

DMCs due to genotype (*App*$^{NL\text{-}G\text{-}F}$ vs WT, control diet) and diet (*App*$^{NL\text{-}G\text{-}F}$ PCS vs control diet) at all ages, controlling for age were compared. Number of overlapping DMCs due to genotype and diet in **A)** cortex and **B)** hippocampus. DMCs required to have $q < 0.05$ in both treatment groups. Heatmaps showing reversal of genotype-dependent methylation by diet in **C)** cortex and **D)** hippocampus. DMGs were determined by mapping each CpG to their nearest gene according to parameters previously discussed. Overlap between DMGs in the **E)** cortex and **F)** hippocampus. Percent change for each DMG was calculated by averaging the percent change for each DMC. Heatmaps showing reversal of genotype-dependent net gene methylation by diet in **G)** cortex and **H)** hippocampus. **I)** Example of how diet can reverse genotype dependent methylation of chr8:46540075, located upstream of *Ascl1,* in the cortex at 9 months. There is a decrease in methylation in *App*$^{NL\text{-}G\text{-}F}$ mice on the control diet (Holm-Sidak adjusted $p = 0.0343$), with PCS reversing genotype changes (Holm-Sidak adjusted $p = 0.0085$). **J)** Example of how diet can reverse genotype dependent methylation of chr10:94052312, located near *Fgd6,* in the hippocampus. Hypomethylation due to *App*$^{NL\text{-}G\text{-}F}$ genotype was reversed by PCS.

Figure S14: Distal DMR Associated with AD Genotype is Enriched for Repressive Histone Marks

A) Example of a DMR that is distally correlated with the expression of microglial inflammation genes **B)** Magnification of region containing *C4b, Aif1, Tnf, Tap1,* and *Psmd9* **C)** Association between *C4b* mRNA and methylation of chr17 39844008, located within a distal DMR **D)** Diagram generated by CATlas (http://catlas.org/catlas_hub/) using data from Zhu et al. 2021. Peak enrichment of histone marks H3K27ac, H3K27me3, and H3K4me1 are shown in hippocampal CA1 neurons, astrocytes, and microglia.

Figure S15: Expression CpGs in *App*$^{NL\text{-}G\text{-}F}$ mice are located within differentially methylated regions

DMRs were generated as described in the methods. **A)** Representative 634 bp DMR within *Tmem267* differentially methylated in *App*$^{NL\text{-}G\text{-}F}$ mice and is correlated with *Tmem267* expression. **B)** HOMER enrichment of DMRs in *App*$^{NL\text{-}G\text{-}F}$ mice reveal ETS family transcription factor sequences over-represented in DMRs. RNA expression of differentially expressed ETS transcription factors in *App*$^{NL\text{-}G\text{-}F}$ mice **C)** cortex and **D)** hippocampus.

Figure S16: Study Design
Seven days prior to mating, female mice were placed on either a choline supplemented or standard diet and maintained until postnatal day 21, when pups were weaned and placed on the control diet. At 3-, 6-, 9-, and 12-months mice were sacrificed and RNA and DNA was extracted from the cortex and hippocampus and underwent RNA-Seq and RRBS.

File S1: Common DMGs between human AD and $App^{NL\text{-}G\text{-}F}$ mice cortex and hippocampus and GO enrichment of common DMGs

File S2: List of DMCs and DMGs altered in AD ("Genotype" group) and reversed by PCS ("Diet" group). DMG percent changes were calculated by averaging individual DMCs located within each gene.

File S3: List of regression statistics between DMCs and their closest differentially expressed gene based on region and treatment group.

File S4: List of regression statistics between DMCs and differentially expressed genes located on the same chromosome based on region and treatment group.

File S5: List of DMRs constructed from expression-associated CpGs based on region and treatment group. Average change calculated by geometric mean of individual CpGs within a region. Correlated genes determined by genes whose expression is associated with one or more individual CpG within the region.

File S6: HOMER enrichment of AD-associated DMRs. Consensus sequences, P-values, and percent of regions containing motifs of interest.

File S7: List of plaque-associated CpGs (PACs) by regression statistics and genes whose RNA is associated with PAC methylation.

File S8: List of CpGs whose association with amyloid depends on PCS diet and genes whose RNA is associated with PAC methylation.
